# Green and Low‐Carbon Synthesis of Lithium Sulfide (Li_2_S): From Fundamental Mechanisms to Scalable Manufacturing for Next‐Generation Energy Storage

**DOI:** 10.1002/advs.76836

**Published:** 2026-07-28

**Authors:** Aiping Peng, Xingtao Qi, Lingfeng Zhu, Ningrui Zhan, Yaohui Qu, Hui Li, Hai Zhang, Fan Wang, Ze Zhang, Xuewen Wang, Zhenyu Yang, Tianyi Ma

**Affiliations:** ^1^ School of Chemistry and Chemical Engineering Nanchang University Nanchang China; ^2^ School of Biological and Chemical Engineering Zhejiang University of Science and Technology Hangzhou China; ^3^ School of Science RMIT University Melbourne Victoria Australia; ^4^ Jiangxi Provincial Key Laboratory of Green Hydrogen and Advanced Catalysis School of Physics Jiangxi Normal University Nanchang Jiangxi China

**Keywords:** green and low‐carbon chemistry, lithium sulfide synthesis, process intensification and scale‐up, sustainable energy materials

## Abstract

Lithium sulfide (Li_2_S) is a critical cathode material for high‐energy‐density lithium sulfur batteries and an indispensable precursor for sulfide solid electrolytes. Traditional high‐temperature carbothermal reduction remains energy‐intensive and carbon‐heavy, creating a significant mismatch with industrial sustainability targets. This review focuses on engineering‐oriented green synthesis routes, systematically analyzing low‐temperature solid‐state reactions, magnesiothermal reduction, and solution‐based metathesis pathways. It highlights process intensification, scale‐up feasibility, techno‐economic analysis, and by‐product valorization. Current challenges in industrial amplification, including mass transfer limitations and cost competitiveness, are critically evaluated. Future directions emphasize continuous‐flow manufacturing, closed‐loop solvent recovery, and hybrid process integration, providing actionable technical pathways to enable cost‐effective, low‐carbon Li_2_S production for industrial applications.

## Graphical abstract

This review focuses on green synthesis routes for lithium sulfide (Li_2_S)—a key material for lithium–sulfur batteries and sulfide solid electrolytes, whose traditional carbothermal reduction remains energy‐intensive and unsustainable. Specifically examined routes include low‐temperature solid‐state reactions, magnesiothermal reduction, and solution‐based metathesis, with emphasis on process intensification, scale‐up feasibility, techno‐economic analysis, and by‐product valorization. Next, challenges in industrial scaling, such as mass transfer and cost, are critically evaluated. Finally, future directions—continuous‐flow manufacturing, closed‐loop solvent recovery, and hybrid process integration—are proposed to enable low‐carbon, cost‐effective Li_2_S production for practical applications.

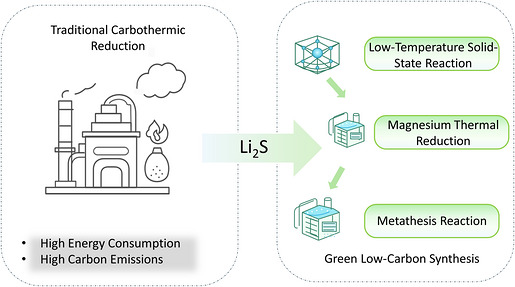

## 1 Introduction

### 1.1 Strategic Position of Li_2_S in Clean Energy Technologies

**FIGURE 1:**
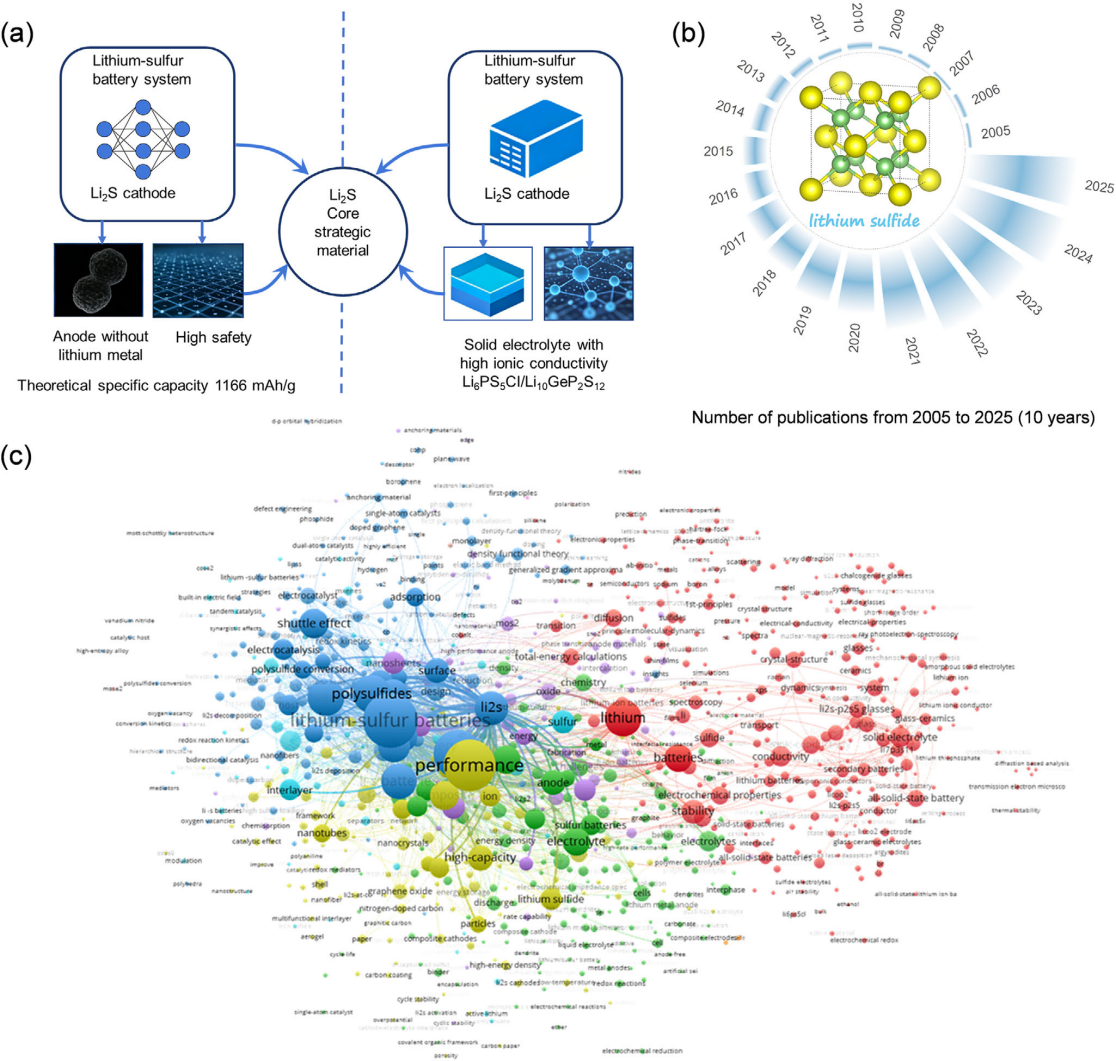
(a) Schematic illustration of the key applications of Li_2_S in lithium‐sulfur batteries and all‐solid‐state batteries. (b) Statistical summary of reported research on lithium sulfide to date. (c) Research hotspots retrieved using lithium sulfide as keywords, data from Web of Science.

Lithium sulfide (Li_2_S) serves as the pivotal cathode active material in lithium‐sulfur (Li‐S) battery systems, offering a remarkable theoretical specific capacity of up to 1166 mAh·g^−1^ (Figure 1a) [1, 2, 3, 4]. It also features intrinsic safety without metallic lithium and pre‐lithiation properties compatible with lithium‐free anodes. Consequently, it is widely acknowledged as a viable candidate for next‐generation high‐energy‐density energy storage systems [5, 6]. Research concentrating on the application and modification of Li_2_S has witnessed a significant upsurge globally (Figure 1b). Compared with conventional elemental sulfur cathodes, Li_2_S enables direct assembly of full‐cell configurations with lithium‐metal‐free anodes. This not only enhances the system energy density but also significantly improves battery operational safety [7, 8, 9, 10]. Additionally, Li_2_S is a critical precursor for synthesizing high‐performance sulfide solid electrolytes, such as Li_6_PS_5_Cl and Li_10_GeP_2_S_12_ [11, 12, 13]. Its chemical purity and crystallinity directly govern the ionic conductivity and interfacial stability of solid electrolytes, thereby emerging as a core raw material that limits the performance of all‐solid‐state batteries [14, 15, 16]. A literature search was carried out on Li_2_S in the Web of Science database, with certain keywords presented in Figure 1c. Subjects associated with Li‐S batteries and sulfide solid electrolytes have garnered substantial research interest.

In recent years, breakthroughs have been continuously achieved in the structural design of Li_2_S‐based composite cathodes. Composite Li_2_S with graphene, porous carbon matrices, or catalytic functional materials can effectively mitigate its inherent drawbacks, including poor intrinsic electronic conductivity, a high initial activation voltage, and the polysulfide shuttle effect [17, 18, 19, 20]. Studies have demonstrated that the Li_2_S/graphene composite cathode (active material loading: 2.2 mg·cm^−2^) fabricated using green and low‐carbon synthesized Li_2_S delivers stable charge‐discharge profiles and superior coulombic efficiency in solvated ionic liquid electrolyte systems [7]. Uniformly dispersing Li_2_S with a particle size of approximately 4 nm in a nanocarbon matrix and compounding it with Li_6_PS_5_Cl solid electrolyte enables a reversible capacity of 830 mAh·g^−1^ at room temperature, corresponding to a Li_2_S utilization rate of 71% [14]. These findings unequivocally validate that the controllable synthesis of high‐purity Li_2_S lays a critical material foundation for promoting the commercialization of Li‐S batteries and advancing all‐solid‐state battery technologies, granting it an irreplaceable strategic status in the clean energy industrial chain [21, 22]. However, Li_2_S should not be regarded as a universal product for different battery applications, because its use as a cathode active material in Li−S batteries and as a precursor for sulfide solid electrolytes imposes markedly different requirements on chemical purity, crystallinity, particle size, morphology, specific surface area, and impurity tolerance.

### 1.2 Environmental and Economic Challenges of the Traditional Carbothermal Reduction Method

Currently, the industrial large‐scale preparation of Li_2_S is predominantly dependent on the high‐temperature carbothermal reduction method. This approach employs lithium sulfate (Li_2_SO_4_) as the raw material, which reacts with carbon sources (e.g., carbon black, glucose) at 800–1000°C to generate Li_2_S, with the simultaneous release of CO or CO_2_ gases [23, 24, 25]. Although this method exhibits high technical maturity and excellent scalability, its intrinsic environmental and economic drawbacks have become increasingly prominent in the context of the global transition to green, low‐carbon, and sustainable development. First, the high‐temperature reaction regime entails massive energy consumption and intensive greenhouse gas emissions, which run counter to the global carbon neutrality target. Second, the reaction system generates complex by‐products, including unreacted carbon sources, lithium carbonate, and sulfur oxides, which limit the product purity and necessitate additional purification steps, further increasing production costs and causing resource waste [26, 27, 28]. Third, the type and properties of carbon sources not only affect the product morphology and electrochemical performance but also introduce uncontrollable impurities that may degrade the interfacial stability of subsequent battery systems [29, 30]. Finally, the process has a low atom economy and a low lithium recovery rate, failing to comply with the basic principles of green chemistry (Table 1).

**TABLE 1:** Comparison of key performance between the traditional high‐temperature carbothermal method and green synthesis processes for Li_2_S.

Synthetic route	Reaction temperature	Direct process CO_2_	Main By‐products	Product purity/Raw material cost	Greenness condition	Hidden energy/pollution burden	Overall low‐carbon potential
**Carbothermal reduction**	Very high, 800–1000°C, inert amosphere	High (CO/CO_2_)	Carbon residue, Li_2_CO_3_, sulfur oxides	Medium/High	Difficult to decarbonize without clean heat/carbon capture	High furnace energy, inert gas demand, purification burden	Low
**Low‐temperature solid‐state reaction**	Low to moderate, <200°C, air or mild atmosphere	Low direct CO_2_ emission	Li_2_SO_3_, L_i2_S_2_O_3,_ H_2_O or sulfur‐containing gases	High/Extremely low	Requires high Li conversion and intermediate recycling	Mechanical activation, incomplete conversion, gas treatment, and lithium recovery	Medium to high
**Magnesiothermal reduction**	Low to moderate, inert atmosphere	No direct carbon‐derived CO_2_ during the main reaction	MgO	High/Medium	Low‐carbon only if the Mg/MgO loop is closed	Mg production/regeneration energy, MgO separation, washing/purification	Medium to high
**Metathesis reaction**	Low, Room temperature, inert atmosphere	No direct carbon‐derived CO_2_ during the main reaction	NaCl, Na_2_SO_4_, residual solvent	Ultra‐high/Low	Low‐carbon only with high solvent/salt recovery	Solvent use, distillation energy, salt separation, wastewater control	High if closed‐loop recovery is achieved

With the tightening of global environmental regulations and the rising requirements for sustainable manufacturing, the traditional carbothermal reduction method is facing dual challenges of environmental pressure and cost competition in large‐scale production, making it imperative to develop efficient, low‐carbon, and green alternative synthetic routes.

### 1.3 Necessity of a Green Synthetic Paradigm: From Carbon Neutrality to Process Innovation

Toward the national “dual carbon” strategic goals and the rapid development of the clean energy materials industry, developing a new green, low‐carbon, and efficient synthetic paradigm for Li_2_S has become a core research topic of common concern to both academia and industry [31, 32]. The core implication of green synthesis encompasses not only the reduction or elimination of hazardous byproducts, decreased reaction energy consumption, and minimized full‐life‐cycle carbon footprint, but also the balanced optimization of atom economy, raw material renewability, and process scalability [23, 26].

In recent years, a variety of innovative green synthesis strategies have been reported successively, providing feasible solutions for the sustainable preparation of Li_2_S (Table 1). On the one hand, non‐carbon reduction routes such as magnesiothermal reduction can complete the reaction rapidly at low temperatures; the by‐product MgO is relatively benign and potentially recyclable, enabling the main reduction step to proceed without direct carbon‐derived CO_2_ emissions [23, 33]. On the other hand, solution‐based synthetic routes based on metathesis reactions (e.g., the reaction of lithium salts with Na_2_S in an ethanol medium) can construct a closed‐loop continuous production system through “out‐of‐solvent crystallization” and solvent vacuum distillation recovery technology, approaching near‐zero waste discharge when efficient solvent recovery and salt by‐product recycling are implemented. The resulting high‐purity Li_2_S product can be directly used for the preparation of battery cathodes and solid electrolytes [26, 27].

The aforementioned emerging synthesis processes show clear reaction‐level advantages over conventional carbothermal reduction; however, their full‐process environmental benefits depend on precursor selection, reducing‐agent regeneration, solvent recovery, by‐product circularity, and purification efficiency. Through rational reaction design and closed‐loop process integration, these routes may improve lithium resource utilization and product uniformity while reducing the overall carbon footprint of Li_2_S manufacturing, providing technical support for the large‐scale, low‐cost, and sustainable supply of Li_2_S [28, 34]. Therefore, promoting the transformation of Li_2_S synthesis from the traditional high‐carbon‐emission and high‐energy‐consumption model to a green and low‐carbon new paradigm is not only an inevitable requirement for fulfilling environmental responsibilities but also a key measure to ensure the safety of the industrial chain of Li‐S batteries and solid‐state batteries and enhance the core competitiveness of the industry [35, 36].

### 1.4 System Boundary for Green and Low‐Carbon Assessment of Li_2_S Synthesis

A reduced reaction temperature or the absence of in‐reactor CO_2_ evolution does not automatically guarantee a genuinely green and low‐carbon Li_2_S manufacturing route. For a rigorous comparison, the sustainability of Li_2_S synthesis should be evaluated within a cradle‐to‐gate boundary, covering not only the main chemical reaction but also upstream precursor production, reducing‐agent preparation or regeneration, solvent consumption, inert‐gas use, mechanical activation, product separation, washing, drying, solvent recovery, and by‐product treatment [37, 38]. This boundary‐aware perspective is particularly important for emerging non‐carbothermal routes.

For low‐temperature solid‐state reactions, the main furnace energy demand is significantly reduced compared with high‐temperature carbothermal reduction; however, incomplete conversion, excess sulfur, oxygen‐containing lithium intermediates, and possible sulfur‐containing gaseous species may introduce additional purification and recovery burdens. For magnesiothermal reduction, the main reduction step avoids carbon‐derived CO_2_ formation, but the embedded energy and emissions associated with metallic Mg production or MgO‐to‐Mg regeneration must be included when evaluating its full‐process carbon footprint. Similarly, solution‐based metathesis proceeds under mild conditions and can deliver high‐purity Li_2_S through precipitation or solvent‐induced crystallization, yet its environmental advantage depends strongly on solvent recovery efficiency, distillation energy, salt by‐product crystallization, and wastewater management [34, 39].

Therefore, “green Li_2_S synthesis” should be regarded as a conditional and system‐level concept rather than a reaction‐level label. A route can be considered truly low‐carbon only when direct process emissions are minimized while indirect environmental burdens from raw materials, solvents, purification, and by‐products are effectively controlled through closed‐loop process integration. This framework provides the basis for the following comparison of low‐temperature solid‐state, magnesiothermal, and metathesis routes.

## 2 Fundamental Reaction Mechanisms of Li_2_S Synthetic Chemistry

### 2.1 Thermodynamic Limitations and Kinetic Defects of High‐Temperature Carbothermal Reduction

**FIGURE 2:**
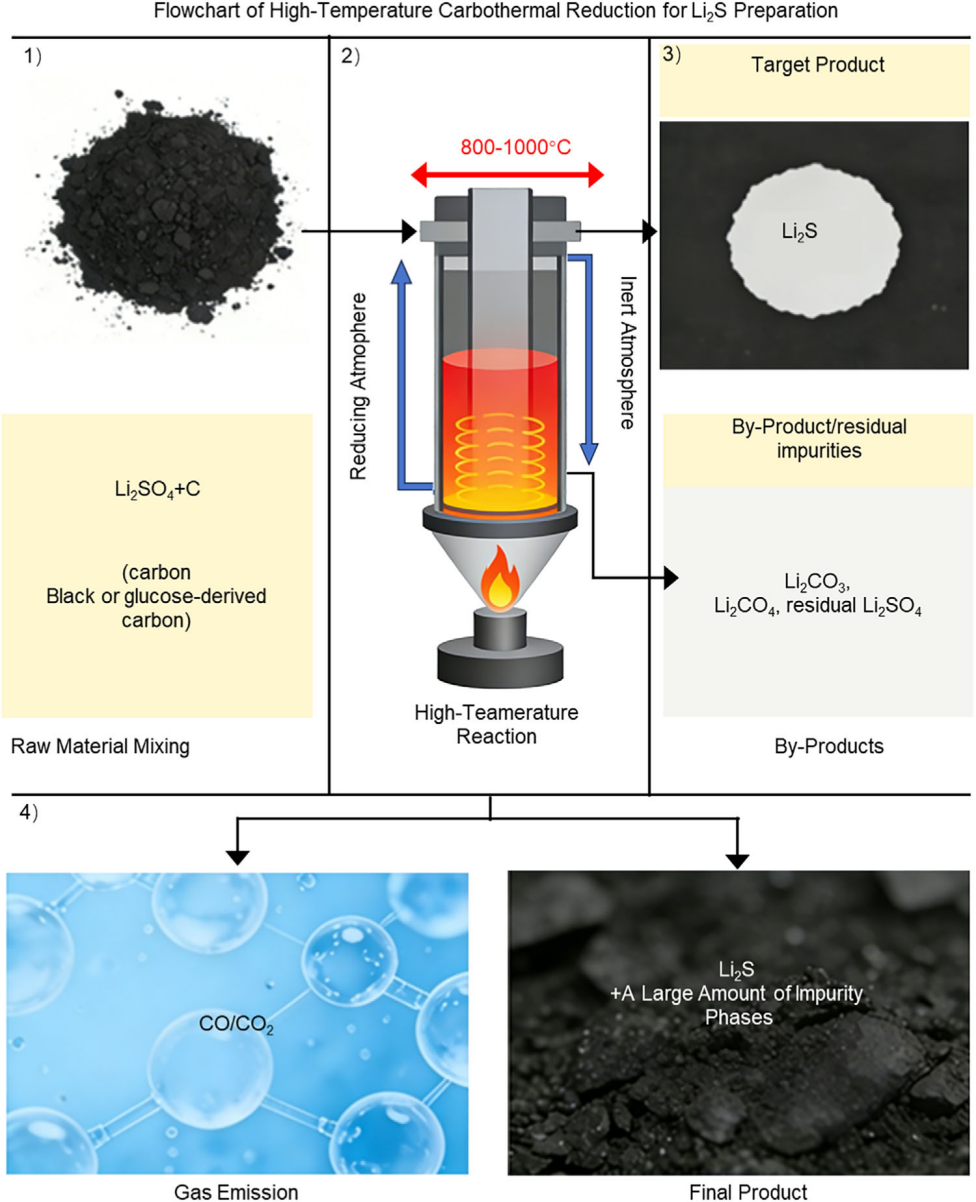
Schematic illustration of the reaction pathway and by‐products for the preparation of Li_2_S via high‐temperature carbothermal reduction of Li_2_SO_4_.

Conventional Li_2_S synthesis predominantly relies on high‐temperature carbothermal reduction under an inert atmosphere. In this process, Li_2_SO_4_ is blended with a carbon precursor and heated to 800–1000°C, where carbon acts as the reducing agent to achieve sulfur valence state reduction. This method has insurmountable inherent defects in both thermodynamic and kinetic aspects (Figure 2).

From a thermodynamic standpoint, the decomposition of Li_2_SO_4_ requires overcoming a high Gibbs free energy barrier, and the reducing capacity of carbon is highly constrained by the reaction temperature window. This inevitably induces side reactions, such as the formation of Li_2_CO_3_ and Li_2_O [40]. From a kinetic perspective, the solid‐solid reaction interface has a limited contact area, which leads to slow reactant mass transfer and a prolonged reaction cycle. Moreover, high‐temperature conditions are prone to cause the volatilization loss of lithium, reducing the lithium recovery rate. Meanwhile, the process emits large amounts of CO/CO_2_ greenhouse gases, which are inconsistent with the development requirements of carbon neutrality [23].

More importantly, Li_2_S products synthesized via the carbothermal route tend to be coated with a thin insulating oxide layer (e.g., Li_2_SO_3_, Li_2_SO_4_, Li_2_CO_3_) on their surface, which significantly increases the initial charge activation barrier of Li_2_S and directly degrades its electrochemical reactivity [41, 42]. These inherent shortcomings have forced researchers to gradually abandon the traditional high‐temperature route and turn to the development of greener and milder low‐temperature alternative synthetic routes.

### 2.2 Pathway Analysis and By‐Product Regulation of the Low‐Temperature Solid‐State Reaction (LiOH‑S System)

**FIGURE 3:**
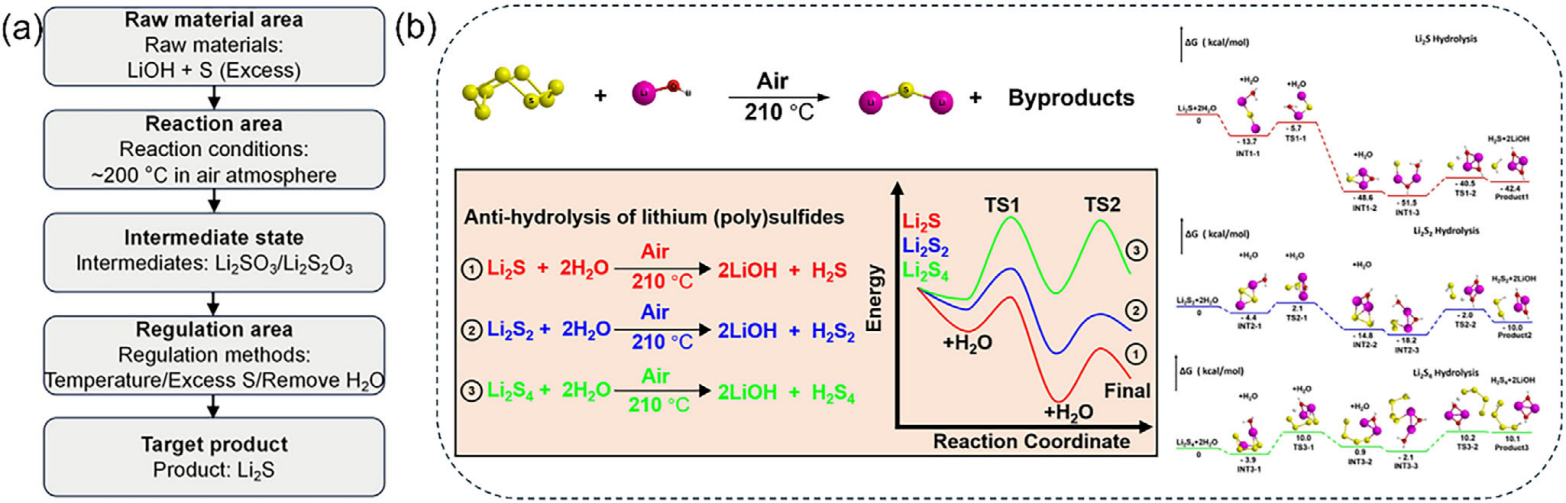
(a,b) Schematic illustration of the reaction pathway and intermediate transformation for the low‐temperature solid‐state synthesis of Li_2_S in the LiOH‑S system. Reproduced with permission from [43]. Copyright 2023 American Chemical Society.

The low‐temperature solid‐state reaction system using lithium hydroxide (LiOH) and elemental sulfur (S) as raw materials represents an important breakthrough in the field of green Li_2_S synthesis in recent years. This reaction can proceed steadily at approximately 200°C in ambient air without inert atmosphere protection, and the dominant reaction pathway is expressed as follows: 2LiOH + S → Li_2_S + H_2_O↑ (Figure 3a). Introducing excess elemental sulfur into the reaction system not only drives the chemical equilibrium to shift toward Li_2_S formation but also effectively inhibits the Li_2_S hydrolysis side reaction. At the same time, the water vapor generated by the reaction is discharged promptly to avoid the occurrence of the reverse reaction [43].

Studies have shown that this reaction pathway undergoes the formation and transformation of intermediate products such as lithium sulfite (Li_2_SO_3_) and lithium thiosulfate (Li_2_S_2_O_3_. Density functional theory (DFT) calculation results reveal that Li_2_S itself has a certain hydrolysis resistance stability, while Li_2_SO_3_ can be further decomposed to regenerate Li_2_S under heating conditions, providing a theoretical possibility for the recycling of by‐products. By systematically optimizing key parameters such as the sulfur‐lithium molar ratio, heating rate and holding time, the lithium conversion rate can be increased to approximately 57%, while a high‐purity Li_2_S product is obtained (Figure 3b). This method greatly reduces the reaction energy consumption and equipment requirements, with the raw material cost reduced to approximately $64.9 per kilogram, less than 10% of the cost of commercial Li_2_S products [43]. Notably, such low‐temperature solid‐state reactions are highly dependent on the micromixing state and interfacial contact efficiency of the precursors. Mechanical activation methods such as planetary ball milling can create microscale “reaction hotspots” in the system, induce a local high‐temperature and high‐pressure environment at room temperature, and effectively promote the initiation and progress of the reaction [44].

### 2.3 Comparison of Electron Transfer Mechanisms for Non‐Carbon Reducing Agents (Mg/Na_2_S)

**FIGURE 4:**
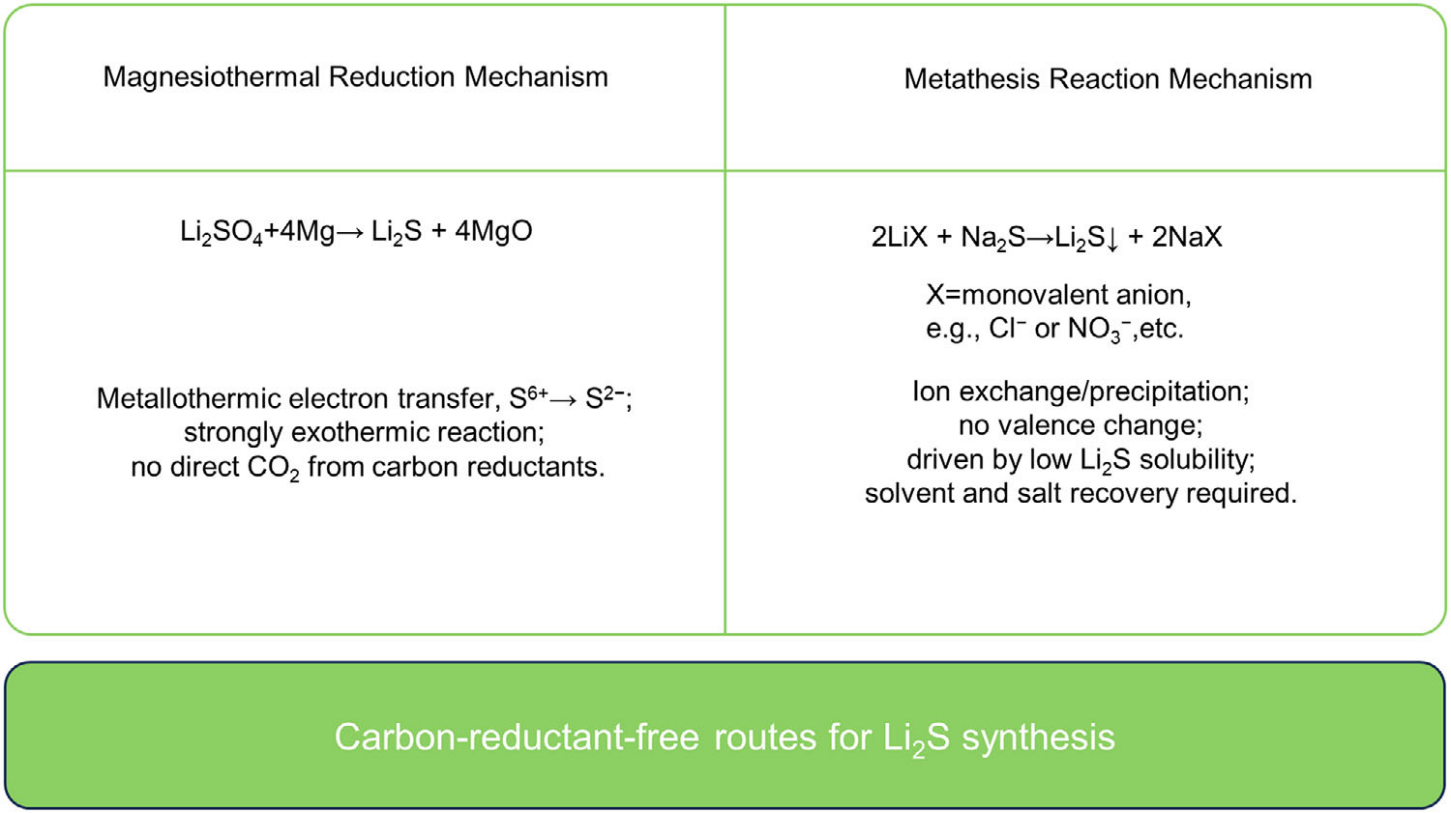
Schematic comparison of reaction‐level decarbonization mechanisms for Li_2_S synthesis via magnesiothermal reduction and metathesis reaction. The absence of carbon‐derived CO_2_ in the main reaction does not exclude indirect upstream or downstream environmental burdens.

To completely avoid the use of carbon sources from the source, non‐carbon reduction strategies have developed rapidly, among which the magnesiothermal reduction method and the lithium salt‐Na_2_S metathesis reaction method are the most representative, and there are essential differences between the two in terms of reaction mechanism and electron transfer pathway (Figure 4).

The magnesiothermal reduction reaction uses metallic magnesium as a strong reducing agent (E° = −2.37 V vs. standard hydrogen electrode (SHE)), which can directly reduce Li_2_SO_4_ to synthesize Li_2_S rapidly, with the reaction being: Li_2_SO_4_ + 4Mg → Li_2_S + 4MgO. This reaction is characterized by instantaneous exothermicity and can be completed at a relatively low temperature without generating any greenhouse gases during the process, with MgO as the by‐product [23]. In the reaction process, electrons are directly transferred from Mg atoms to the sulfur center, realizing the multi‐electron reduction of S^6+^→S^2−^. The reaction kinetics are fast, and the resulting product has high crystallinity, making it suitable for solvent‐free dry synthesis. Nevertheless, the CO_2_‐free feature of the core magnesiothermal reaction does not represent a genuine zero‐carbon fabrication pathway. Metallic magnesium is a highly energy‐intensive reducing agent, and its upstream production or regenerative recycling from MgO by‐products contributes substantial indirect carbon emissions, especially when fossil fuel‐based power and heat are adopted. Additionally, subsequent MgO separation, washing, and purification inevitably consume water and acidic reagents, potentially generating secondary waste. Consequently, the low‐carbon advantages of magnesiothermal reduction can be fully realized only when integrated with a closed‐loop Mg/MgO recycling system, renewable electricity supply, or high‐value by‐product utilization strategies.

**FIGURE 5:**
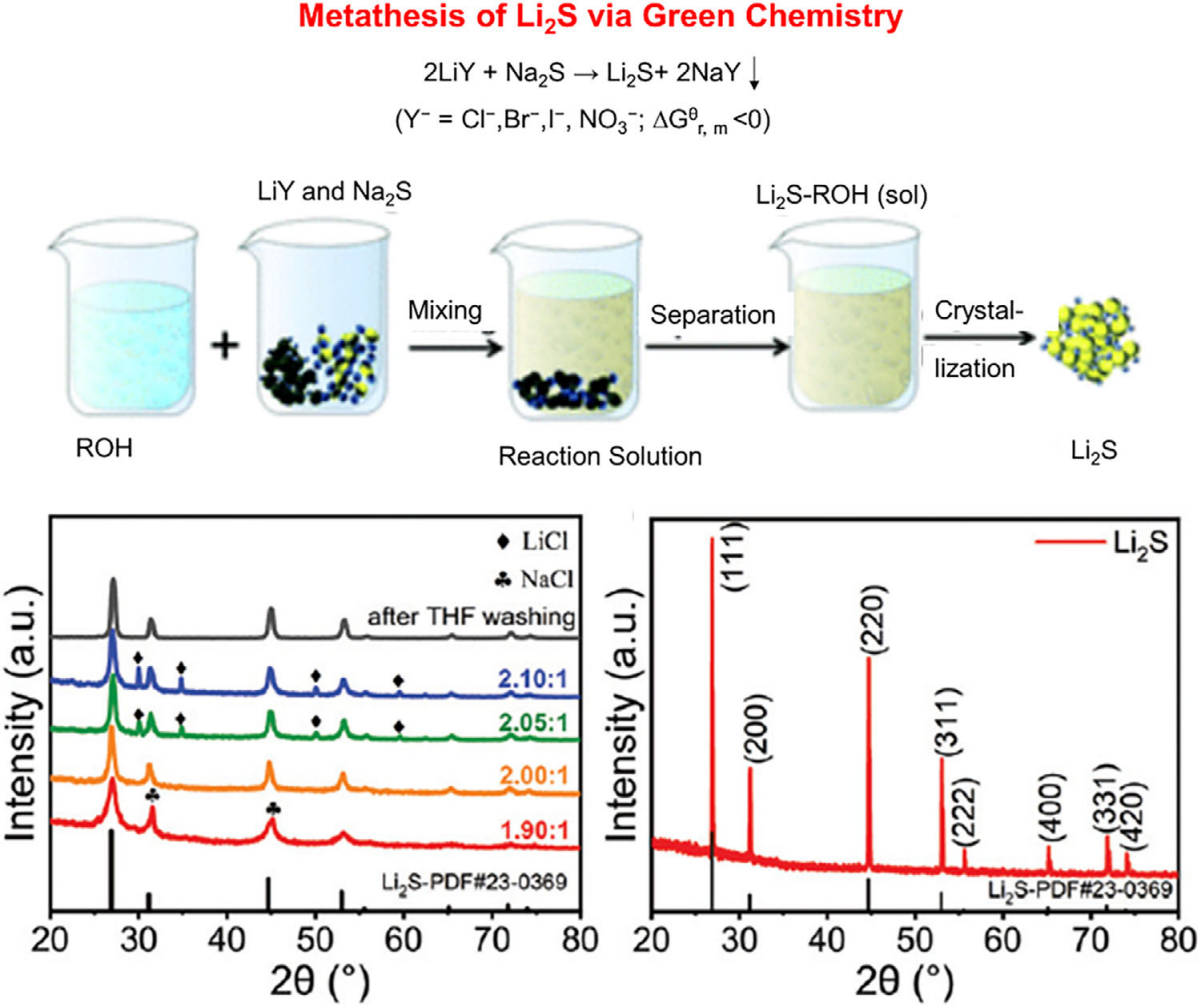
Schematic comparison of Li_2_S synthesis mechanisms and X‐ray diffraction (XRD) of Li_2_S from LiCl/Na_2_S ratios (P1.90−P2.10) and P2.10 after THF washing. Reproduced with permission from [45]. Copyright 2022 The Royal Society of Chemistry.

The metathesis reaction between lithium salts and Na_2_S follows an ion exchange mechanism, with the reaction being: 2LiX + Na_2_S → Li_2_S↓ + 2NaX (X = halogen ion or NO_3_^−^) (Figure 5). This reaction can proceed spontaneously in room‐temperature organic solvents, and the thermodynamic driving force originates from the extremely low solubility product (K_sp_ ≈ 10^−25^) of Li_2_S. High‐purity Li_2_S crystals can be directly precipitated through “out‐of‐solvent crystallization” technology [26, 45]. This reaction involves no electron transfer and is only an ion rearrangement process, with extremely low reaction energy consumption. In addition, the solvent can be recovered in a closed loop via vacuum distillation, truly achieving zero waste discharge and making it suitable for continuous solution‐phase production. Both non‐carbon reduction strategies are significantly superior to the traditional high‐temperature carbothermal method in terms of environmental friendliness and economic potential. For solution‐based metathesis, the main environmental burden is shifted from high‐temperature reaction energy to solvent and salt management. Organic solvents such as THF or alcohols require high recovery efficiency to avoid volatile organic compound emissions and excessive distillation energy. Meanwhile, soluble by‐products such as NaCl or Na_2_SO_4_ must be removed through washing, crystallization, or membrane‐based separation, which may generate wastewater if not operated in a closed loop [46]. Thus, metathesis routes should be described as potentially low‐waste rather than intrinsically waste‐free, and their practical sustainability depends on solvent circularity, salt recovery, and product‐separation efficiency [47].

### 2.4 Multiphase Reaction Mechanisms and Interfacial Transport Limitations

Beyond reaction stoichiometry and macroscopic process descriptions, Li_2_S synthesis should be regarded as a series of multiphase transformations governed by interfacial contact, mass transport, heat transfer, and phase evolution. This mechanistic perspective is particularly important for low‐temperature solid‐state reactions, metallothermic reduction, and solution‐based metathesis. Although these routes are thermodynamically feasible under specific conditions, their multiphase nature can lead to non‐uniform conversion and kinetic limitations that cannot be fully explained by thermodynamic analysis alone [3, 4].

For low‐temperature solid‐state synthesis, the LiOH−S route is a typical heterogeneous solid−solid/solid−gas reaction system. Efficient Li_2_S formation requires intimate contact between lithium‐ and sulfur‐containing precursors, continuous renewal and replenishment of reactive solid interfaces, and timely removal of volatile by‐products such as H_2_O when generated. Mechanical activation can promote these processes by reducing particle size, creating fresh contact surfaces, introducing lattice defects, and generating local high‐reactivity regions. Nevertheless, the overall reaction kinetics remain sensitive to precursor micromixing, particle packing, spatial heat distribution, and solid‐state diffusion across intermediate phases [47, 48]. Under insufficient mixing or prolonged reaction conditions, sluggish interface renewal and diffusion may result in unreacted particle cores, spatially heterogeneous lithium conversion, and accumulation of oxygen‐containing intermediates such as Li_2_SO_3_ and Li_2_S_2_O_3_. These intermediate species may partially cover active interfaces and block interparticle mass‐transport pathways, thereby hindering complete and uniform Li_2_S formation.

For metallothermic reduction, represented by magnesiothermal and aluminothermal routes, Li_2_S formation involves stronger multiphase coupling among solid precursors, metallic reductants, possible transient liquid/vapor transport, and in situ generated oxide products. The reaction is initiated at discrete contact interfaces between the metallic reductant and lithium/sulfur‐containing precursor, accompanied by rapid local heat release. As the reaction proceeds, oxide by‐products such as MgO or Al_2_O_3_ may accumulate around reactive particles and form product layers [49, 50]. These layers can physically isolate unreacted precursors from fresh reductants and restrict further interfacial contact. Therefore, the apparent reaction rate is controlled not only by thermodynamic driving force and interfacial electron transfer, but also by reductant diffusion, transport resistance through product layers, local heat accumulation/dissipation, and dynamic regeneration of active reaction interfaces [51].

In contrast, solution‐based metathesis is governed by liquid‐phase mass transfer coupled with rapid precipitation and crystallization kinetics. The low solubility of Li_2_S in suitable solvent systems provides a strong driving force for precipitation. However, ion exchange and nucleation may occur before complete macroscopic mixing is achieved, generating localized supersaturation domains at the microscale. Once the local supersaturation exceeds the nucleation threshold, burst nucleation, particle aggregation, and entrapment of soluble inorganic salts may occur [52, 53]. These crystallization events directly affect Li_2_S particle‐size distribution, crystallinity, residual salt content, and product purity. Therefore, the key mechanistic parameters for metathesis routes include micromixing intensity, feeding sequence and rate, local supersaturation distribution, nucleation rate, crystal‐growth kinetics, and the dynamic composition of the mother liquor.

Overall, the intrinsic mechanisms of green Li_2_S synthesis are not determined solely by reaction temperature or idealized reaction equations. Instead, they are jointly regulated by multiphase interfacial transport, product‐layer evolution, gaseous by‐product removal, spatial heat distribution, and microscopic crystallization kinetics. Clarifying these mechanistic factors provides a fundamental basis for subsequent process optimization, reactor design, and scalable Li_2_S manufacturing.

## 3 Technological Breakthroughs in Low‐Temperature Solid‐State Synthesis

### 3.1 Design of Air‐Stable Reaction Systems (<200°C)

To address the traditional carbothermal reduction method's reliance on inert atmospheres and high energy consumption, researchers have focused on developing new Li_2_S synthetic routes that can operate reliably in air at temperatures below 200°C. Notably, the solid‐state reaction system using LiOH and elemental sulfur as raw materials offers significant advantages. This method leverages the chemical stability of LiOH and S in air, enabling their direct reaction to form Li_2_S at temperatures below 200°C. An inert carrier gas is introduced to promptly remove the by‐product water vapor, thereby effectively suppressing Li_2_S hydrolysis [43]. The use of excess elemental sulfur in the reaction not only improves the raw material conversion rate but also forms a protective layer on the surface of product particles, further enhancing the structural stability of Li_2_S in humid air.

**FIGURE 6:**
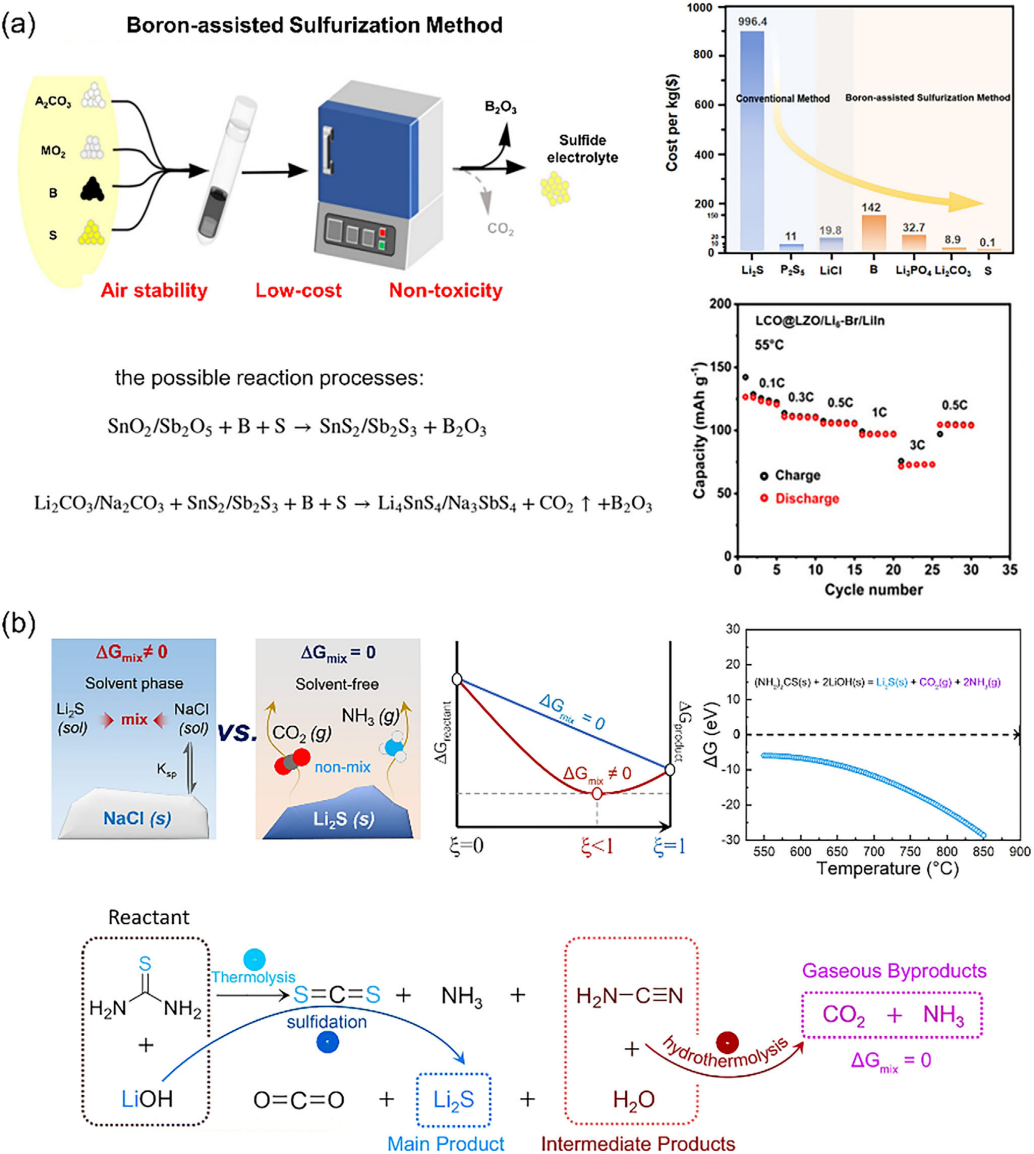
(a) The synthesis processes of the boron‐assisted sulfurization method, the raw materials cost of Li_6_PS_5_Cl, and the rate performance of Li_6_‐Br. Reproduced with permission from [54]. Copyright 2025, American Chemical Society. (b) Thiourea‐assisted solvent‐free metathesis for scalable preparation of Li_2_S. Reproduced with permission from [37]. Copyright 2025, Springer Nature.

In addition to the LiOH‑S system, boron‐assisted sulfidation has also been successfully applied to the low‐temperature synthesis of various sulfide solid electrolytes (Figure 6a). Its core principle is that boron atoms preferentially combine with oxygen atoms in oxide precursors to form volatile or inert by‐products, releasing active sites for sulfur to participate in the reaction. The entire process can be completed at temperatures below 200°C without a strictly water‐ and oxygen‐free reaction environment [54]. Another type of solvent‐free synthesis strategy uses thiourea as the sulfur source to react with LiOH at temperatures below 200°C (Figure 6b). Intermediates (HNCN, H_2_O) can be spontaneously converted in situ into harmless gases such as CO and NH_3_, which escape from the system, continuously driving the reaction toward Li_2_S formation and avoiding the problem of organic solvent residue [37]. This strategy has the potential for kilogram‐scale industrial amplification.

### 3.2 Optimization of Transformation Pathways for Intermediates (Li_2_SO_3_/Li_2_S_2_O_3_)

In the process of low‐temperature solid‐state synthesis of Li_2_S in the LiOH‑S system, oxygen‐containing intermediates such as lithium sulfite (Li_2_SO_3_) and lithium thiosulfate (Li_2_S_2_O_3_) are often generated as by‐products. If these intermediates cannot be efficiently converted, the purity and reaction yield of Li_2_S will be significantly reduced. Studies have confirmed that Li_2_SO_3_ can undergo disproportionation or reductive decomposition reactions under specific thermodynamic conditions to regenerate the target product Li_2_S. This phenomenon has been verified by DFT theoretical calculations, revealing the intrinsic regeneration mechanism of oxygen‐containing intermediates [43].

To promote the complete conversion of intermediates to Li_2_S, researchers have proposed three pathway optimization strategies. First, the efficient transformation of intermediates such as Li_2_SO_3_ to Li_2_S is driven by precisely regulating the reaction temperature gradient, extending the holding time, and optimizing the excess ratio of elemental sulfur. Second, in the boron‐assisted sulfidation system, the strong oxygen affinity of boron atoms is used to preferentially capture oxygen atoms in the system, inhibiting the accumulation of Li_2_SO_3_ and simultaneously promoting more efficient participation of sulfur in the sulfidation reaction, reducing the residue of oxygen‐containing by‐products [54]. Third, the solvent‐free thiourea synthesis route continuously breaks the reaction equilibrium through the in situ generation of volatile by‐products, making it difficult for intermediates to exist stably, thus realizing the directional and controllable preparation of high‐purity Li_2_S [37]. The core idea of these strategies is consistent: achieving the irreversible transformation of oxygen‐containing intermediates to the target product Li_2_S through thermodynamic driving or kinetic intervention.

### 3.3 Balancing Atom Economy Improvement and Lithium Recovery Rate

Green synthesis not only pursues mild reaction conditions but also emphasizes maximizing resource utilization efficiency and atom economy. In the low‐temperature solid‐state synthesis system of Li_2_S, the selection of lithium sources and the method of by‐product management directly determine the lithium recovery rate and the overall process sustainability. Taking the LiOH‑S reaction system as an example, although this method has low raw material costs, only approximately 57% of LiOH conversion was achieved in initial studies, with nearly half of the lithium resources not being effectively utilized [43].

To improve atom economy, subsequent studies have significantly enhanced the lithium conversion efficiency by precisely controlling the reaction material ratio, introducing catalytic additives, and optimizing the heat treatment procedure. Among them, the solvent‐free route using thiourea as the sulfur source has obvious advantages: this method does not consume organic solvents, and the by‐products are spontaneously escaping gases (CO, NH_3_), which can theoretically achieve a sulfur utilization rate close to 100%. At the same time, almost all lithium atoms enter the Li_2_S crystal lattice, greatly improving the overall atom economy. This method has been successfully applied to the preparation of kilogram‐scale sulfide solid electrolytes (Li_6_PS_5_Cl, Li_10_GeP_2_S_12_), reducing the laboratory‐level production costs by 27.5% and 92.9%, respectively, and demonstrating excellent resource efficiency and economic feasibility [37]. In the future, coupling the recovery process of unreacted LiOH and lithium in by‐products to construct a full‐process closed‐loop production system will be a key direction for achieving the synergistic improvement of lithium recovery rate and synthesis efficiency. For low‐temperature solid‐state routes, the recovery of lithium‐containing intermediates and the capture of possible sulfur‐containing gaseous species are essential for improving atom economy and preventing secondary pollution.

## 4 Innovative Progress in Non‐Carbon Reduction Strategies

### 4.1 Transient Reaction Characteristics and Zero‐Carbon Emission Advantages of Magnesiothermal Reduction

Magnesiothermal reduction is a typical non‐carbon reduction route for the preparation of Li_2_S, which has exhibited multiple advantages in the field of green synthesis in recent years: (1) This method can realize the rapid synthesis of Li_2_S at a relatively low temperature, and the reaction process is characterized by significant instantaneous exothermicity, which greatly shortens the reaction cycle and improves synthesis efficiency. (2) Unlike the traditional carbothermal reduction method that relies on carbon sources and emits large amounts of CO_2_, the magnesiothermal reduction method does not contain carbon‐based reducing agents, thus avoiding greenhouse gas generation from the source and achieving true zero carbon emissions [23]. (3) The reaction by‐product (MgO) has stable chemical properties, is easy to separate, and can be used as a functional additive to improve the cycle stability of battery cathode materials [55]. The practical sustainability of this route, however, depends on whether MgO can be separated with sufficient purity and redirected into high‐value applications or a Mg/MgO regeneration loop.

In addition, the magnesiothermal reduction method has been successfully verified in other oxide reduction systems such as SiO_2_, confirming its good universality in low‐temperature and efficient reduction. Li_2_S prepared by this method exhibits excellent electrochemical performance when applied in Li‐S batteries, including good cycle stability, a low initial activation voltage, and outstanding rate charge‐discharge capability, further highlighting the dual advantages of the magnesiothermal reduction method in material function regulation and environmental friendliness [23].

### 4.2 Thermodynamically Spontaneous Characteristics of the Lithium Salt‐Sodium Sulfide Metathesis Reaction

In addition to metal thermal reduction, the metathesis reaction between lithium salts and sodium sulfide (Na_2_S) constitutes another important non‐carbon reduction strategy for synthesizing Li_2_S. The core principle of this route is to exploit ion exchange between lithium salts (e.g., halides, nitrates, sulfates) and Na_2_S in solution or solid phase to directly produce Li_2_S precipitates [45]. Due to the low solubility and high thermodynamic stability of the product Li_2_S, the entire reaction can proceed spontaneously at room temperature or under moderate heating conditions, without external energy input or strong reducing agents [26].

**FIGURE 7:**
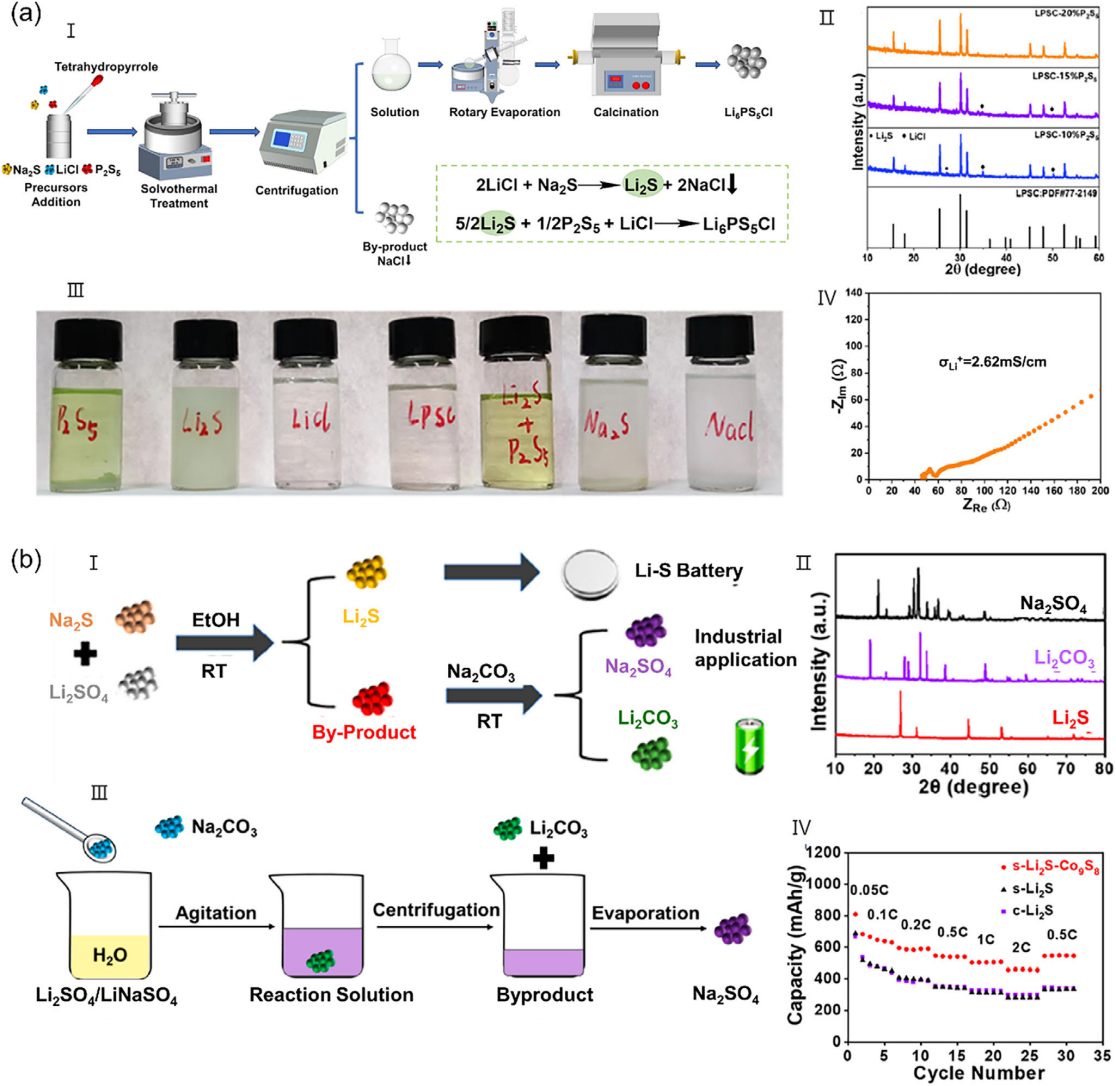
(a) Methodological development of solvothermal synthesis of LPSC (Li_6_PS_5_Cl) from Li_2_S, P_2_S_5_, and LiCl using THP as the solvent, and performance assessments of the synthesized material in terms of conductivity. Reproduced with permission from [56]. Copyright 2024 Royal Society of Chemistry. (b) Schematic illustration of the four‐stage process for Li_2_S preparation from Li_2_SO_4_ and Na_2_S via metathetic reaction, along with the byproduct recovery flowchart and the rate capability performance. Thiourea‐assisted solvent‐free metathesis for scalable preparation of Li_2_S. Reproduced with permission from [27]. Copyright 2024, American Chemical Society.

Studies have shown that the reaction of LiCl with Na_2_S in a tetrahydrofuran solvent can efficiently prepare high‐purity Li_2_S intermediates, which can be directly used for the subsequent synthesis of sulfide solid electrolytes such as Li_6_PS_5_Cl (Figure 7a) [56]. The reaction of Li_2_SO_4_ with Na_2_S in an ethanol medium simplifies the product separation process by utilizing the low solubility of Li_2_SO_4_, and simultaneously converts the by‐product LiNaSO_4_ and unreacted raw materials into high‐value‐added materials, realizing the cascade and efficient utilization of resources (Figure 7b) [27]. Notably, such metathesis reactions are typically conducted in organic solvents. When combined with low‐boiling anti‐solvent‐based “solvent‐induced crystallization” technology, vacuum distillation‐based solvent recovery and closed‐loop continuous production can be achieved, significantly enhancing process economics and sustainability [26]. In addition to solvent recovery, closed‐loop salt management is required because NaCl, Na_2_SO_4_, or mixed lithium−sodium salts generated in the mother liquor may otherwise become secondary waste streams.

### 4.3 Correlation between Reducing Agent Selection and Product Purity

The chemical properties of reducing agents and their interaction mechanisms with sulfur sources directly determine the reaction selectivity of Li_2_S synthetic routes and the final product purity. In the magnesiothermal reduction system, the strong reducing ability of metallic magnesium can efficiently drive the reaction forward; however, improper control of reaction conditions may cause over‐reduction or side reactions, affecting the product phase purity. After optimizing the reaction parameters (precisely controlling the magnesium dosage, reaction temperature, and holding time), a high‐purity Li_2_S product can be obtained, and the by‐product MgO can be easily removed by water washing or acid washing [23].

In contrast, the metathesis reaction route involves no electron transfer process and has a “cleaner” reaction pathway. Product impurities are mainly derived from coexisting ions in the raw materials, thus imposing high requirements on the initial purity of lithium salts and Na_2_S. Studies have found that anions of different lithium salts have a significant impact on the reaction efficiency and product morphology, and the halide system is more conducive to obtaining Li_2_S products with good crystallinity [45]. In addition, the physical and chemical properties of by‐products (e.g., solubility, thermal stability) directly determine the design of post‐treatment processes: MgO is an insoluble solid, facilitating separation by filtration; soluble salts generated by metathesis reactions, such as NaCl and Na_2_SO_4,_ need to be removed by washing or recrystallization. Therefore, the selection of reducing agents or reaction raw materials is not only related to the smooth progress of the reaction but also profoundly affects the product purity, process complexity, and overall atom economy, which needs to be comprehensively optimized in the design of green synthetic routes.

## 5 Advanced Characterization Techniques and Mechanism Research

### 5.1 In Situ XRD/Raman for Dynamic Monitoring of Solid‐Solid Phase Transitions

**FIGURE 8:**
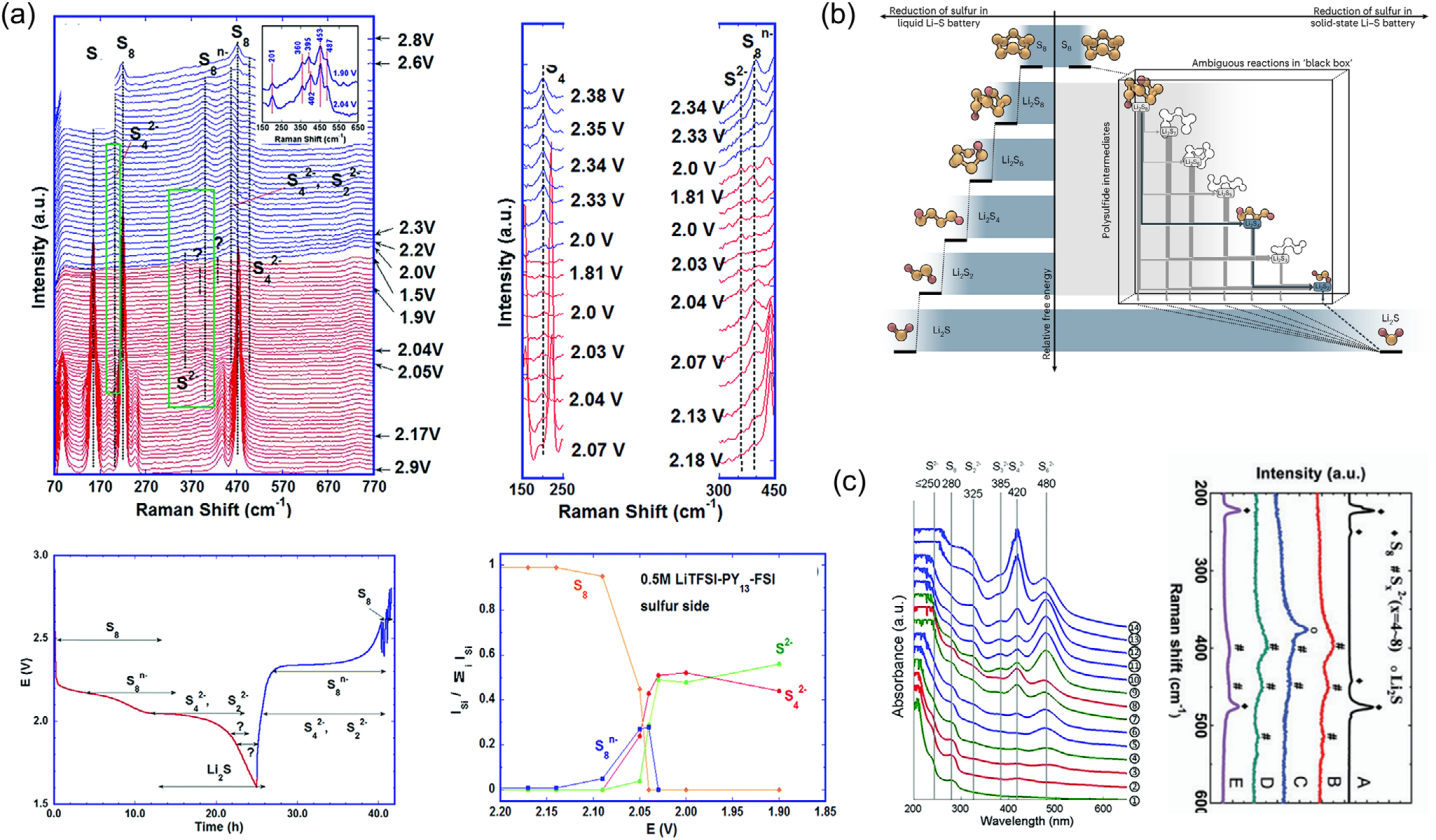
(a) Raman spectra of a Li−S cell at C/48: spectral evolution, zoomed view, polysulfide species (red: discharge, blue: charge), and intensity ratio vs. potential. Reproduced with permission from [58]. Copyright 2017 The Royal Society of Chemistry. (b) Mapping the conversion pathway of sulfur in liquid and all‐solid‐state configurations. Reproduced with permission from [60]. Copyright 2024 Springer Nature. (c) Schematic illustration of monitoring Li_2_S synthesis and electrochemical reaction phase transitions via in situ Raman spectroscopy. Reproduced with permission from [58]. Copyright 2017 The Royal Society of Chemistry.

In the process of Li_2_S synthesis and electrochemical reaction transformation, the dynamic evolution mechanism of solid‐solid phase transitions has long been limited by the lack of in situ characterization techniques with high spatiotemporal resolution. In recent years, the rapid development of in situ X‐ray diffraction (XRD) and Raman spectroscopy technologies has provided key tools for real‐time tracking of reaction pathways. During the discharge process of Li‐S batteries, in situ XRD combined with electrochemical test results reveals that Li_2_S nanocrystalline nuclei begin to nucleate at approximately 2.0 V and rapidly deposit into a lamellar structure at 1.83 V; during the charging stage, only a portion of Li_2_S is oxidized and dissolved, and the continuous accumulation of residues is the direct cause of battery capacity fading (Figure 8a) [57, 58].

In situ Raman spectroscopy can effectively monitor the transformation process of polysulfide intermediates such as Li_2_S_4_ and Li_2_S_6_ to the final product Li_2_S and identify differences in reaction pathways under different current densities (Figure 8b,c) [59, 60]. The aforementioned characterization techniques can not only capture the formation and disappearance processes of intermediate phases such as Li_2_SO_3_ and Li_2_S_2_O_3_ but also quantitatively analyze their stability windows in low‐temperature solid‐state reactions, providing a reliable experimental basis for the precise regulation of by‐products.

### 5.2 Theoretical Calculation‐Guided Prediction of Reaction Energy Barriers

Theoretical calculations, especially first‐principles simulations based on density functional theory (DFT), are core tools for predicting the reaction energy barriers and thermodynamic feasibility of Li_2_S synthetic routes [61, 62]. High‐throughput DFT calculations combined with the machine learning interatomic potential (MLIPs) calculation framework can accurately identify the stable products and their spatial distribution at the lithium metal/solid electrolyte interface, such as nanocrystalline Li_2_S regions formed in the amorphous interphase layer [63]. Simulation results reveal that the interfacial reaction can usually be divided into two stages: rapid initial diffusion and slow subsequent phase transition.

In addition, the cross‐correlation effect of Li‐P and P‐S ion motion significantly affects the phosphorus diffusion behavior, which may lead to the kinetic trapping of the Li‐P phase and thus limit the interfacial ion transport efficiency [64]. In non‐carbon reduction systems, theoretical calculations are used to evaluate the electron transfer activation energy when Mg or Na_2_S is used as the reducing agent, confirming that the magnesiothermal reduction pathway has a lower reaction energy barrier, which is consistent with its experimentally observed instantaneous exothermic characteristics.

Furthermore, molecular dynamics simulations show that the long‐range electrostatic interaction between active centers and polysulfides can promote the formation of dense phases rich in Li and S (2≤n≤6), and realize the instantaneous deposition of non‐equilibrium Li_2_S through a collective charge transfer mechanism [17]. These calculation results provide a priori guidance for the design of experimental schemes and effectively shorten the process optimization cycle.

### 5.3 Effect of Interfacial Contact Engineering on Reaction Efficiency

In the process of solid‐phase synthesis of Li_2_S, the physical contact state between reactant particles directly determines the transmission efficiency of electrons and ions, thereby affecting the overall reaction rate and product purity. Studies have confirmed that poor solid‐solid interfacial contact can lead to problems such as local reaction stagnation, by‐product accumulation and decreased lithium utilization [65]. Therefore, interfacial contact engineering has become a key strategy to improve the efficiency of low‐temperature solid‐state synthesis.

Three‐dimensional nanoarray (3D‐NAs) platforms have ordered, continuous and fully exposed active surfaces, which can efficiently promote mass transfer and charge transfer between reactants and provide an ideal microenvironment for solid‐phase reactions [66, 67]. In instantaneous exothermic reactions such as magnesiothermal reduction, regulating the mixing uniformity and particle size of precursors can optimize the interfacial contact area between Mg and Li_2_SO_4,_ improve the reduction efficiency and inhibit the formation of unreacted cores. Notably, interfacial engineering is not limited to the improvement of physical contact but also includes the regulation of chemical compatibility [68]. For example, when Li_2_S is used for the preparation of solid electrolytes, the in situ construction of a LiCl‐based framework or an amorphous Ti‐S‐P‐Cl interfacial phase can simultaneously improve the ionic conductivity and interfacial stability of the electrolyte. Therefore, the quality of interfacial contact is a core factor determining whether green synthetic routes can be efficiently implemented.

## 6 Scalable Production and Cost‐Benefit Analysis

### 6.1 Optimization of Process Parameters for Kilogram‐Scale Amplification Experiments

**FIGURE 9:**
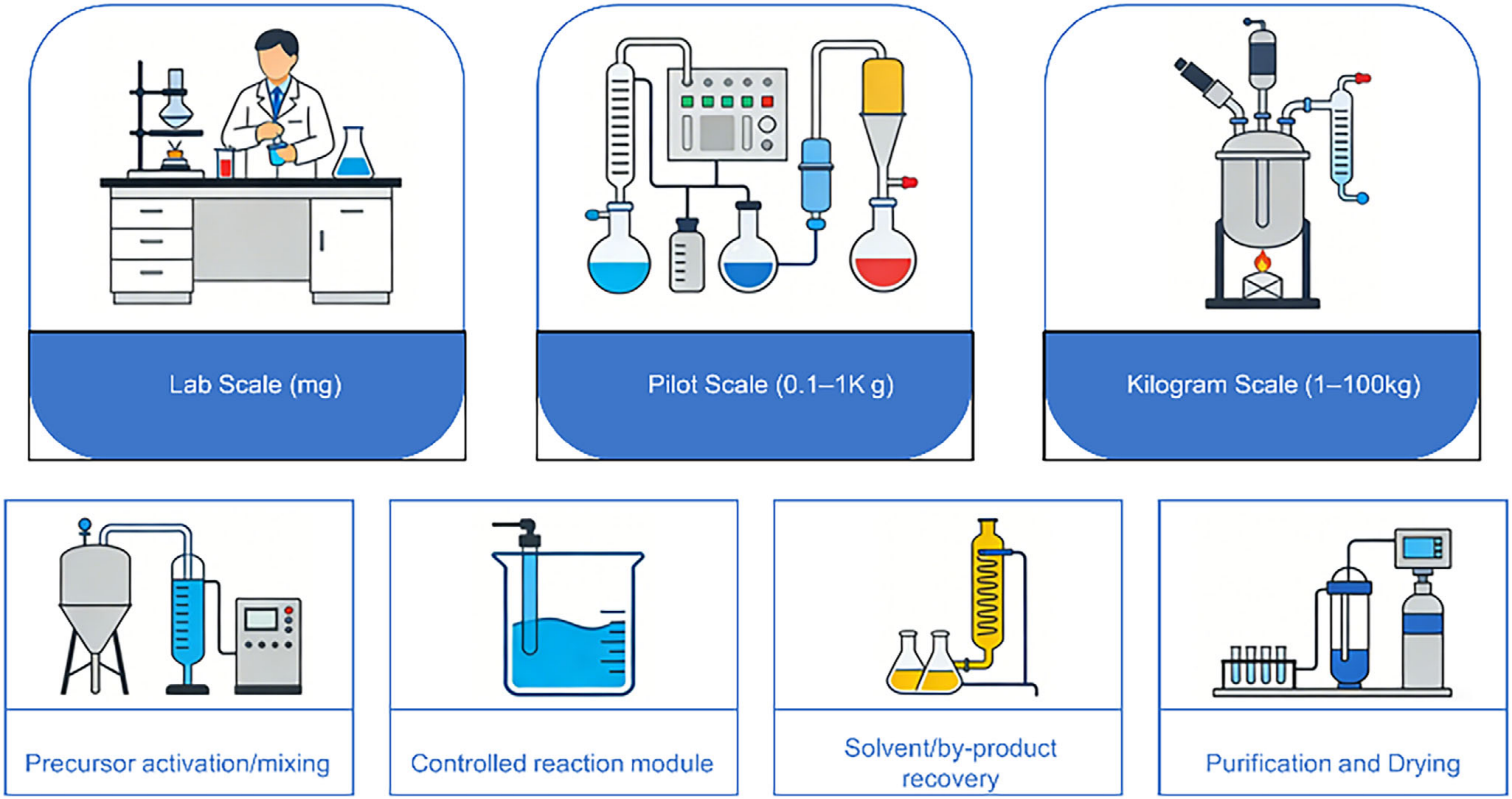
Schematic illustration of the scale‐up process of green Li_2_S synthesis from laboratory to industrialization.

In recent years, several green Li_2_S synthesis strategies have successfully advanced from laboratory‐scale validation to kilogram‐scale amplification (Figure 9). Notably, the solvent‐free route using thiourea as the sulfur source for direct sulfidation of lithium hydroxide has achieved single‐batch synthesis of ∼100 g of Li_2_S and was subsequently scaled up for the preparation of kilogram‐scale sulfide solid electrolytes [37]. This process eliminates the need for organic solvents or inert atmosphere protection and operates under ambient pressure, thus significantly simplifying equipment requirements and operational procedures. At the same time, the intermediates generated during the reaction can be spontaneously converted into harmless gases and escape from the system, effectively driving the reaction toward product formation and improving the process robustness and repeatability.

The metathesis reaction between lithium salts and sodium sulfide realizes closed‐loop continuous production through “solvent‐induced crystallization” combined with vacuum distillation technology, ensuring high product purity while improving the controllability and stability of the process during scale‐up [26]. These studies prove that through the rational design of reaction pathways and post‐treatment steps, the stable and controllable preparation of kilogram‐scale Li_2_S can be achieved without sacrificing product quality.

### 6.2 Magnitude of Cost Reduction Compared with Commercial Li_2_S

Green synthetic routes have shown potential economic advantages for Li_2_S production. When Li_2_S prepared by the solvent‐free metathesis method is used for sulfide solid electrolytes such as Li_6_PS_5_Cl, the overall production cost has been reported to decrease by 27.5%∼92.9% compared with commercially available Li_2_S products [37]. Such cost reduction mainly stems from three factors: first, the optimization of raw material selection, which reduces the cost of basic raw materials; second, the resource utilization of by‐products, which reduces waste treatment costs and creates additional benefits; third, the significant reduction in reaction energy consumption, which lowers operating costs [27, 45]. To make the techno‐economic comparison more quantitative and reproducible, a preliminary raw‐material‐ and electricity‐cost‐normalized estimation was further conducted for representative Li_2_S synthesis routes. In this revised analysis, all costs were normalized to the production of 1 kg Li_2_S, and the consumption of key reagents was recalculated according to the corresponding reaction stoichiometry or corrected using reported yield/conversion values when available [69, 70, 71, 72]. Current supplier quotations were used where possible for lithium sources, sulfur sources, reducing agents, sulfide sources, gases, and solvents, with comparable purity and package size considered to minimize price‐basis inconsistency.

The baseline variable cost was estimated as:

$$C_{\mathrm{variable}}=C_{\mathrm{raw}\;\mathrm{materials}}+C_{\mathrm{gas}}+C_{\mathrm{electricity}}+C_{\mathrm{solvent}/\mathrm{recovery}}$$

where C_raw materials_ represents the stoichiometric or yield‐corrected reagent cost, C_gas_ represents inert‐gas consumption when required, C_electricity_ represents the electricity cost of major unit operations, and C_solvent/recovery_ represents solvent input or net solvent loss after recovery. The electricity cost was calculated from the rated power, operating time, and electricity price of the main processing steps, including ball milling, heating, drying, stirring, solvent evaporation, and condensation:

$$C_{\mathrm{electricity}}=\left[\sum \left(P_{i}\times t_{i}\times f_{i}\right)/m_{Li^{2}S}\right]\times p_{\mathrm{electricity}}$$

where P_i_ is the rated power of equipment i, t_i_ is the operating time, f_i_ is the load factor, m_Li2S_ is the obtained or yield‐corrected mass of Li_2_S, and p_electricity_ is the electricity price. The detailed assumptions, raw‐material prices, reagent amounts, gas/solvent consumption, electricity use, and the raw‐material prices are summarized in Table 2, while the estimated variable costs are presented in Tables 3, 4, 5. It should be noted that this calculation is a preliminary variable‐cost estimation rather than a complete industrial techno‐economic assessment. Equipment depreciation, labor, facility construction, maintenance, purification loss, waste‐treatment cost, and uncertain by‐product credits were not included in the baseline calculation because these terms are strongly dependent on plant scale, process integration, recovery efficiency, and local market conditions. Therefore, the present analysis is mainly intended to provide a transparent and internally consistent comparison of raw‐material and electricity cost contributions among different Li_2_S synthesis routes.

**TABLE 2:** The price of materials.

Item	Average Market price (¥t^−1^)	Update date	Data source
LiOH	140 000	10‐Jun.‐2026	https://new‐energy.smm.cn
Sulfur	12 000	10‐Jun.‐2026	https://new‐energy.smm.cn
Li_2_SO_4_	64 300	10‐Jun.‐2026	https://new‐energy.smm.cn
Mg powder	17 700	10‐Jun.‐2026	https://new‐energy.smm.cn
N_2_ (gas)	2800	10‐Jun.‐2026	https://hz.bendibao.com
LiCl	140 000	10‐Jun.‐2026	https://new‐energy.smm.cn
Na_2_S (66%)	3300	10‐Jun.‐2026	https://new‐energy.smm.cn
EtOH	5755	10‐Jun.‐2026	https://new‐energy.smm.cn
Electricity (Hangzhou)	**¥**1.1/kWh	10‐Jun.‐2026	https://hz.bendibao.com
Exchange rates 1$ = 6.76 ¥ (10‐Jun.‐2026)			

**TABLE 3:** Preliminary variable‐cost estimation for producing 1 kg of Li_2_S via low‐temperature LiOH–S solid‐state synthesis.

Cost category	Item/unit operation	Unit price	Calculation basis	Required amount/operating time	Estimated cost (USD kg^−1^ Li_2_S)	Notes
6LiOH +3S → 2Li_2_S + Li_2_SO_3_ + 3H_2_O						
Raw materials	LiOH	$22.10/kg	Stoichiometric or yield‐corrected amount	1.56 kg	34.47	Corrected by reported Li conversion or product yield
Raw materials	Sulfur	$1.43/kg	Stoichiometric or excess amount	1.1 kg	1.57	Excess sulfur is included if used
Electricity	Ball milling (0.75KW)	Electricity price ($0.162/kWh)	Power × time (10 h)	10 h	1.20	Calculated from rated power and operating time
Electricity	Heating/drying (8KW)	Electricity price ($0.162/kWh)	Power × time (10 h)	10 h	12.96	Normalized to 1 kg Li_2_S
Estimated total	—	—	—		50.20	Raw‐material and electricity costs only

**TABLE 4:** Preliminary variable‐cost estimation for producing 1 kg of Li_2_S via magnesiothermal reduction.

Cost category	Item/unit operation	Unit price	Calculation basis	Required amount/operating time	Estimated cost (USD kg^−1^ Li_2_S)	Notes
Li_2_SO_4_ + 4Mg → Li_2_S + 4MgO						
Raw materials	Li_2_SO_4_	$9.6/kg	Stoichiometric or yield‐corrected amount	2.39	22.94	Lithium source
Raw materials	Mg powder	$2.63/kg	Stoichiometric or excess amount	2.226	5.85	MgO by‐product credit is not directly subtracted.
Gas	N_2_	Supplier quotation (0.41/kg)	Flow rate × time (10 h)	10 h	4.1	If an inert atmosphere is required
Electricity	Heating/drying (5KW)	Electricity price ($0.162/kWh)	Power × time (7 h)	7 h	5.67	Includes initiation heating and post‐treatment drying
Estimated total	—	—	—		38.56	By‐product value is discussed separately.

**TABLE 5:** Preliminary variable‐cost estimation for producing 1 kg of Li_2_S via solution metathesis.

Cost category	Item/unit operation	Unit price	Calculation basis	Required amount/operating time	Estimated cost (USD kg^−1^ Li_2_S)	Notes
2LiCl + Na_2_S → Li_2_S + 2NaCl						
Raw materials	LiCl	$20.7/kg	Stoichiometric or yield‐corrected amount	1.85	38.30	Lithium source
Raw materials	Na_2_S (66%)	$0.488/kg	Stoichiometric or excess amount	2.57	1.25	Sulfide source
Solvent	EtOH	$0.97/L	Net solvent loss or total solvent input	4L	3.88	Solvent recovery efficiency should be specified
Electricity	Stirring/pumping (900 W, 500 W)	Electricity price ($0.162/kWh)	Power × time (1 h, 2 h)	1 h, 2 h	0.31	Route‐dependent
Electricity	Heating/drying (8KW)	Electricity price ($0.162/kWh)	Power × time (24 h)	24 h	30.01	Often a major cost term
Estimated total	—	—	—		73.75	Salt by‐product credit is discussed separately

*Note*: The estimated costs represent preliminary variable costs normalized to the production of 1 kg Li_2_S and include raw materials, gases, solvents, and electricity where applicable. Equipment depreciation, labor, facility construction, maintenance, waste treatment, purification loss, and uncertain by‐product credits are not included.

The analysis further indicates that the economic competitiveness of green Li_2_S synthesis should not be evaluated only by comparing the apparent raw‐material cost or the reported commercial Li_2_S price. For example, converting by‐products such as LiNaSO_4_ or unreacted Li_2_SO_4_ into Li_2_CO_3_ and Na_2_SO_4_ can improve resource utilization, but the additional conversion and purification steps also increase process complexity. Similarly, metathesis reactions can proceed under mild conditions and avoid high‐temperature heating, but solvent recovery, salt separation, and drying may introduce additional cost. In contrast, traditional carbothermal reduction benefits from industrial maturity but requires high‐temperature operation and gas management, leading to persistent energy and emission burdens. Therefore, a reliable techno‐economic comparison should integrate raw‐material consumption, electricity demand, gas/solvent use, purification burden, lithium recovery efficiency, waste‐treatment demand, by‐product credit, and carbon intensity within a unified evaluation boundary.

### 6.3 Techno‐Economic Indicators Linking Low Carbon and Low Cost

Low‐carbon performance and low‐cost feasibility are strongly correlated but not equivalent in Li_2_S manufacturing. Low‐temperature synthesis reduces furnace energy consumption and direct CO_2_ emissions; however, the overall production cost may rise substantially if the process relies on expensive precursors, excessive solvent consumption, complex purification workflows, or inefficient by‐product disposal [73]. Therefore, a unified cradle‐to‐gate system boundary is required for fair comparison, with all metrics normalized to the production of 1 kg of qualified Li_2_S product when data are available.

For standardized techno‐economic evaluation, the key indicators include raw material expenditure, energy input, processing operation, product purification, waste management, lithium recovery efficiency, product yield, solvent recovery efficiency, and by‐product economic credits [74, 75, 76, 77]. The total variable manufacturing cost per kilogram of Li_2_S can be described as:

$$\begin{array}{ccc} \mathrm{Cos}t_{Li^{2}S} & = & C_{\mathrm{raw}}+C_{\mathrm{energy}}+C_{\mathrm{processing}}+C_{\mathrm{purification}}\\ & & +\;C_{\mathrm{waste}}-C_{\mathrm{by}-\mathrm{product}} \end{array}$$

where C_raw_ corresponds to the cost of lithium sources, sulfur feedstocks, reducing agents and organic solvents; C_energy_ accounts for energy consumption during heating, mechanical activation, drying, solvent distillation and inert gas supply; C_processing_ and C_purification_ cover unit operations including precursor mixing, filtration, washing, crystallization and impurity elimination; C_waste_ represents the treatment cost of gaseous, liquid and solid waste streams; C_by‐product_ denotes the economic credits generated from recyclable or marketable by‐products, such as MgO, NaCl, Na_2_SO_4_, and Li_2_CO_3_.

Similarly, the full‐process carbon intensity should integrate both on‐site direct emissions and off‐site indirect carbon burdens:

$$CI_{Li^{2}S}=CI_{\mathrm{direct}}+CI_{\mathrm{energy}}+CI_{\mathrm{precursor}}+CI_{\mathrm{solvent}}+CI_{\mathrm{waste}}$$

where CI_direct_ refers to CO/CO_2_ emissions released during the core reaction; CI_energy_, CI_precursor_, CI_solvent_, and CI_waste_ represent indirect carbon footprints originating from energy supply, upstream precursor production, solvent recovery, and waste treatment, respectively. This indicator‐based framework clarifies that the most competitive green synthesis route is not simply determined by low reaction temperature, but by the comprehensive optimization of raw material affordability, energy efficiency, purification simplicity, carbon intensity, lithium recovery, and closed‐loop by‐product utilization.

Under this indicator system, the three emerging green routes exhibit distinct trade‐offs (Table 6). Magnesiothermal reduction achieves remarkable reaction‐level decarbonization, yet its industrial techno‐economic performance is highly dependent on magnesium raw material pricing, MgO recovery efficiency, and stable Mg/MgO cyclic regeneration. Solution metathesis features mild operating conditions and high Li_2_S selectivity, but its cost and carbon advantages are governed by solvent recovery efficiency, salt by‐product separation, and wastewater closed‐loop control. Low‐temperature solid‐state synthesis minimizes thermal input, while incomplete lithium conversion and insufficient intermediate recycling may increase unit production cost and residual impurity content. Accordingly, future industrial assessment and route comparison should simultaneously adopt unified cost and carbon indicators within a consistent cradle‐to‐gate evaluation boundary.

**TABLE 6:** Key techno‐economic indicators for evaluating green Li_2_S synthesis routes.

Indicator	Definition	Influence on cost	Influence on carbon footprint
**Raw material cost**	Cost of Li sources, S sources, reducing agents, and solvents normalized to 1 kg Li_2_S	Determines baseline production cost	Reflects embodied emissions of precursors
**Energy consumption**	Heating, ball milling, drying, solvent distillation, and inert‐gas operation	Determines operating cost	Determines indirect energy‐related CO_2_ emissions
**Product yield**	Actual Li_2_S obtained relative to the theoretical amount	Low yield increases the cost per kg Li_2_S	Low yield increases emissions per kg Li_2_S
**Lithium recovery efficiency**	Fraction of lithium retained in Li_2_S or recycled streams	Critical because Li‐containing precursors dominate the cost	Reduces lithium waste and associated treatment emissions
**Purification burden**	Washing, filtration, recrystallization, impurity removal, and drying	Adds equipment and operation cost	Adds energy and wastewater‐treatment burden
**Solvent recovery efficiency**	Washing, filtration, recrystallization, impurity removal, and drying	Reduces solvent purchase and disposal costs	Reduces solvent‐related emissions and VOC release
**By‐product credit**	Economic value of MgO, NaCl, Na_2_SO_4_, Li_2_CO_3_, or other recoverable by‐products	Offsets production cost	Reduces waste‐treatment footprint
**Waste‐treatment demand**	Treatment of gas, liquid, or solid waste streams	Adds environmental compliance cost	Adds indirect emissions from waste management

### 6.4 Quantitative Comparison of Energy Consumption and Carbon Footprint

Based on the techno‐economic indicators defined above, the energy consumption and carbon footprint of different Li_2_S synthesis routes should be compared under the same cradle‐to‐gate boundary. In terms of environmental impact, green synthesis strategies exhibit clear reaction‐level low‐carbon advantages compared with traditional methods. However, their environmental benefits should be evaluated by distinguishing direct reaction emissions from indirect cradle‐to‐gate burdens, including precursor production, reducing‐agent regeneration, solvent recovery, product purification, drying, and by‐product treatment [78]. Magnesiothermal reduction can complete the synthesis of Li_2_S rapidly at a relatively low temperature, with no direct carbon‐derived CO_2_ emission during the main reaction. The by‐product MgO can be recycled for functional materials such as battery separator coatings, resulting in a potentially reduced full‐process carbon footprint [23]. Nevertheless, the upstream energy demand for metallic Mg production or MgO regeneration, together with MgO separation and purification, should be considered when assessing the overall sustainability of this route. Aluminothermal reduction also avoids the use of carbon‐based reducing agents; the self‐exothermic characteristic of the reaction further reduces the demand for external energy, and the by‐product Al_2_O_3_ has recycling value [79]. Similarly, its practical low‐carbon advantage depends on the feasibility of high‐value Al_2_O_3_ utilization and closed‐loop by‐product management.

In contrast, the traditional carbothermal reduction method not only consumes a large amount of fossil energy to maintain the high‐temperature reaction but also releases a large amount of CO_2_ due to carbon oxidation, resulting in extremely high carbon emission intensity. Research data show that the synthesis of Li_2_S via solvent‐free metathesis or metathesis precipitation can significantly reduce the energy consumption and carbon emissions per unit product [26, 37]. However, for solution‐based metathesis, solvent distillation, salt separation, washing, and wastewater control may introduce additional energy consumption and environmental burdens if closed‐loop recovery is not achieved. Research data confirm that non‐carbon reduction and low‐temperature solid‐state synthesis routes are not only technically feasible but also have significant advantages in terms of reaction‐level decarbonization. Therefore, their true low‐carbon potential should be assessed under a full‐process framework that considers both direct emissions and indirect burdens, rather than by reaction temperature or in‐reactor CO_2_ release alone.

## 7 Performance Verification of Synthetic Materials in Battery Systems

### 7.1 Ionic Conductivity of Sulfide Solid Electrolytes

Sulfide solid electrolytes such as Li_6_PS_5_Cl and Li_10_GeP_2_S_12_ are regarded as highly promising electrolyte materials for all‐solid‐state batteries due to their high ionic conductivity and good deformability (Table 7). Results from ^7^Li nuclear magnetic resonance (NMR) experiments and DFT molecular dynamics simulations confirm that Li_6_PS_5_Cl exhibits excellent lithium ion transport capacity in the bulk phase [80].

**TABLE 7:** Ionic conductivity of typical sulfide solid electrolytes prepared with Li_2_S as the precursor.

Li source	Electrolyte material	Room‐temperature ionic conductivity	Characteristics	References
LiCl	Li_6_PS_5_Cl	3.3 × 10^−3^ S·cm^−1^	High stability, commonly used	[81]
LiH/Li_2_O	LiPSOCl	2.53 mS·cm^−1^	Oxygen substitution, high conductivity	[82]
Li_2_S	LiSiPS (Co‐doped)	6.91 × 10^−3^ S·cm^−1^	Silicon doping modification	[83]
Li_2_S	LiS‑PS glass‐ceramic	∼10^−2^ S·cm^−1^	Amorphous, high conductivity	[84]

The oxygen‐substituted sulfide solid electrolyte LiPSOCl, synthesized via anionic chemical design, achieves a high ionic conductivity of 2.53 mS·cm^−1^ at room temperature [82]. The LiSiPS electrolyte with a tetragonal structure synthesized by regulating the silicon content has an intrinsic ionic conductivity of 4.28 × 10^−3^ S·cm^−1^, which is further increased to 6.91 × 10^−3^ S·cm^−1^ after doping with 1% Co [83]. The glass‐ceramic LiS‑PS system exhibits a high ionic conductivity on the order of ∼10^−2^ S·cm^−1^ [84]. Despite the excellent bulk ionic conductivity of the aforementioned sulfide electrolytes, the lithium ion transport rate at the interface between Li_6_PS_5_Cl and Li_2_S is significantly lower than the bulk value by several orders of magnitude, which has become a key bottleneck restricting the electrochemical performance of full cells.

### 7.2 Cycle Stability and Interfacial Compatibility of All‐Solid‐State Batteries

**FIGURE 10:**
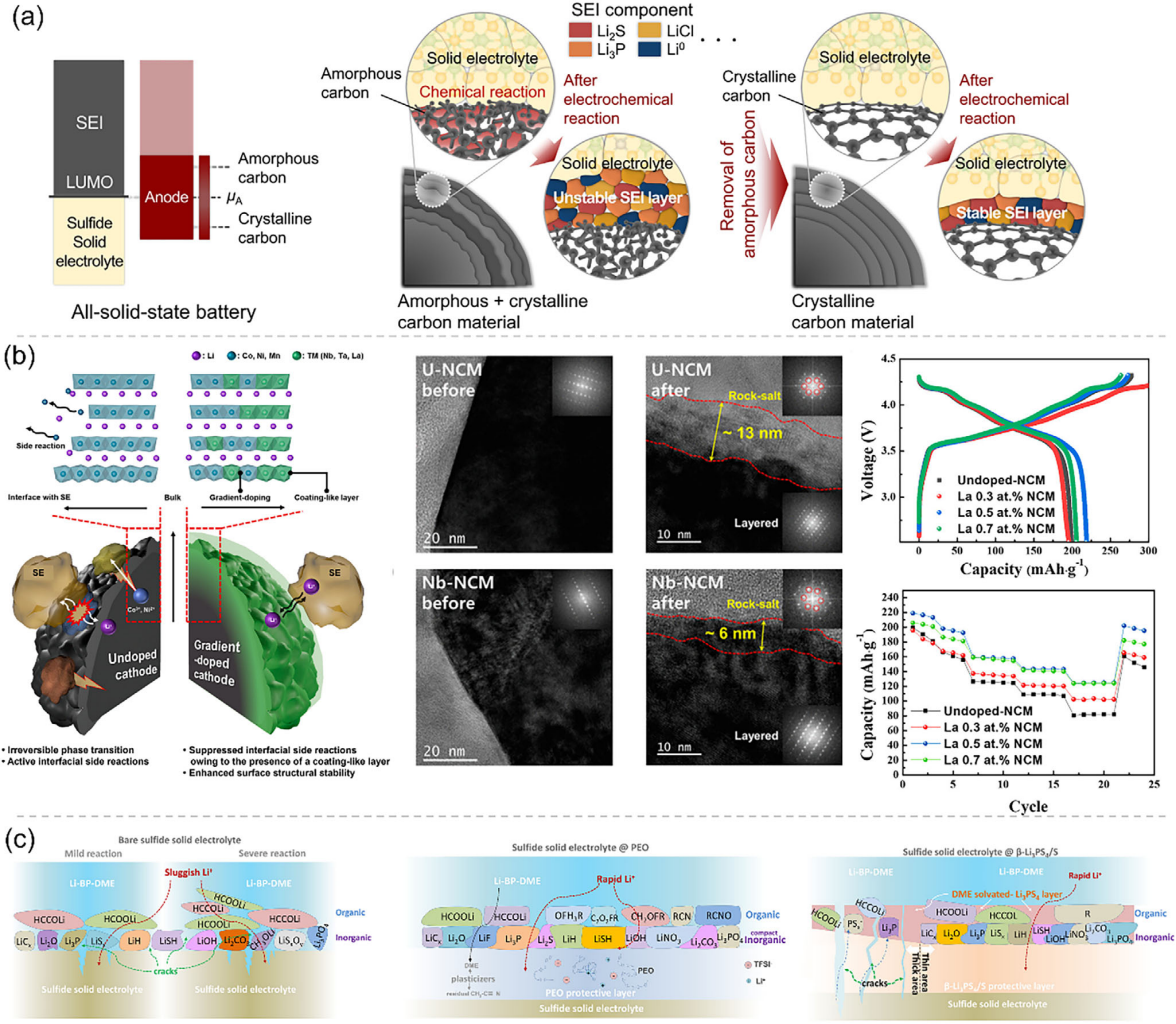
(a) For sulfide solid electrolytes (lowest unoccupied molecular orbital (LUMO) below the anode), amorphous carbon (higher energy level) triggers unstable solid electrolyte interphase(SEI) and high interfacial resistance, whereas crystalline carbon forms a stable SEI and improves electrochemical performance. Reproduced with permission from [87]. Copyright 2024 Elsevier. (b) The effects of gradient doping on all‐solid‐state battery (ASSB) cathodes and their electrochemical performance. Reproduced with permission from [90]. Copyright 2024 American Chemical Society. (c) Schematic illustrations of SEI structures of bare Li_7_P_3_S_11_, poly(ethylene oxide) (PEO)‐lithium bis(trifluoromethanesulfonyl)imide (LiTFSI) interface protective layer, and *β*‐Li_3_PS_4_/S interface protective layer. Reproduced with permission from [91]. Copyright 2023 American Chemical Society.

Although sulfide electrolytes have high ionic conductivity and good mechanical ductility, they are prone to interfacial side reactions when in contact with electrode materials, leading to a continuous increase in interfacial resistance and seriously affecting the cycle stability of batteries (Figure 10a) [85, 86, 87]. To solve this problem, a variety of interfacial engineering strategies have been proposed successively.

On the cathode side, introducing a LilnO_2_‐LiI composite coating between the Li[Ni_0.8_Co_0.15_Al_0.05_]O_2_ cathode and Li_6_PS_5_Cl electrolyte can effectively inhibit the diffusion of sulfur and phosphorus ions to the oxide cathode, significantly reducing the interfacial impedance and improving the rate performance [88]. Surface modification of the 5 V‐class spinel LiNiMnO_4_ anode with 8 wt% LiNbO_3_ can passivate the interface and inhibit the continuous decomposition of the electrolyte, enabling the battery to maintain a reversible capacity of 80 mAh·g^−1^ at the 20th cycle (Figure 10b) [89]. Cathode materials with gradient doping of Nb, Ta, or La oxides can form a surface‐enriched layer, reducing side reactions with sulfide electrolytes, and the interfacial protection effect is comparable to that of traditional coatings [90]. On the anode side, constructing a compatible interfacial protective layer such as PEO or β‐Li_3_PS_4_ can achieve stable cycling for more than 1000 h between the sulfide electrolyte and lithium metal (Figure 10c) [91].

In terms of actual battery performance, all‐solid‐state lithium batteries based on LiPSOCl electrolyte can cycle stably for more than 100 times with a high active material loading of 10.7 mg·cm^−2^ and a total cycle number of over 1000 times [82]. Li_6_PS_5_Cl‐based batteries with a SeS solid solution cathode exhibit a specific capacity of more than 1100 mAh·g^−1^(reaching 98.5% of the theoretical value) at a current density of 50 mA·g^−1^ and cycle stably for 100 times, with a high loading areal capacity of 12.6 mAh·cm^−2^ [92].

### 7.3 Enhancement Mechanism of Li_2_S Decomposition Kinetics by Catalytic Effects

**FIGURE 11:**
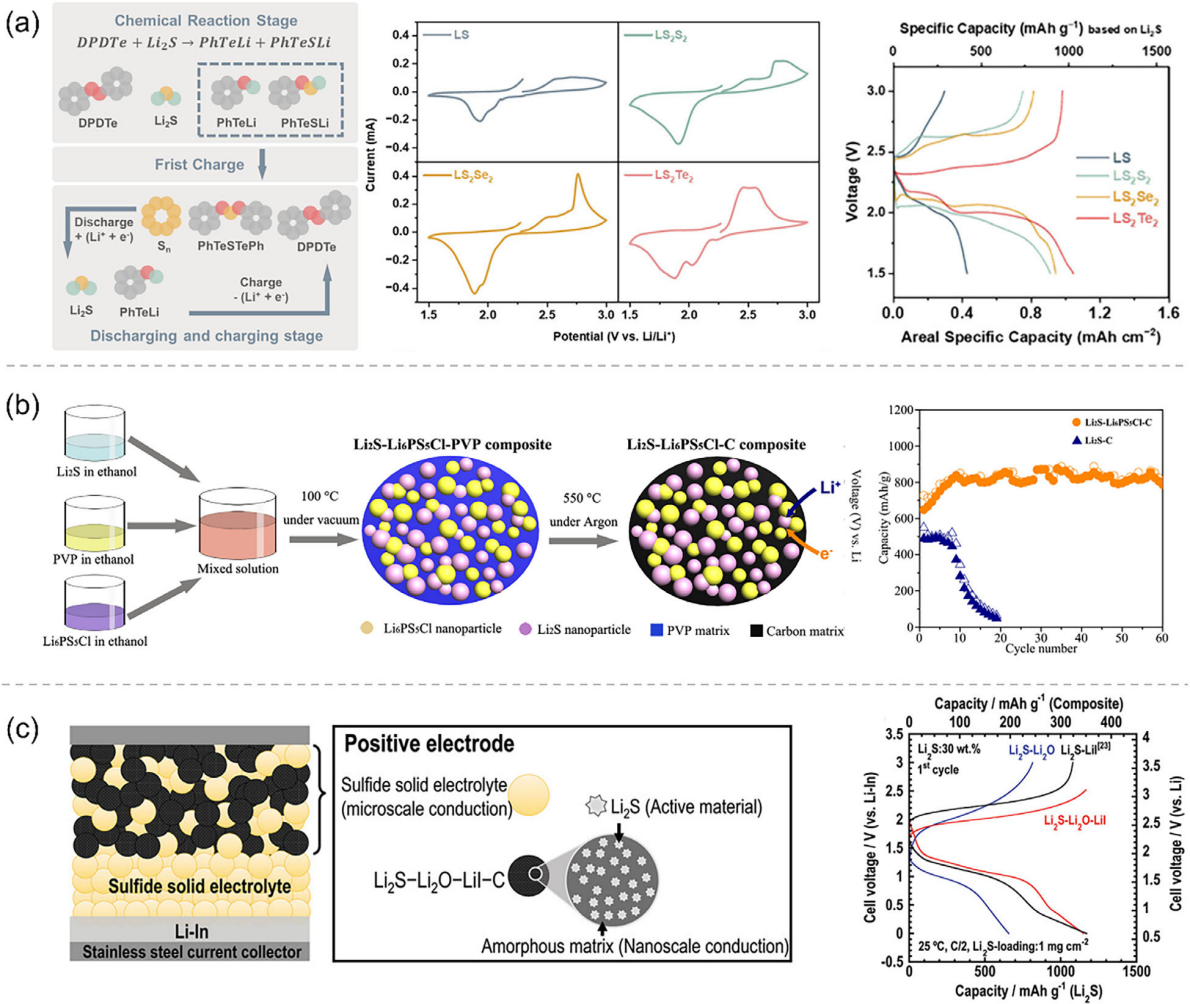
(a) Charge–discharge roadmap of the LS_2_Te_2_ cathode. CVs (0.05 mV s^−1^) and cycling performance (5 cycles at 0.1 mA cm^−2^) of Li_2_S, LS_2_S_2_, LS_2_Se_2_, and LS_2_Te_2_ cathodes. Reproduced with permission from [93]. Copyright 2025, Wiley‐VCH. (b) First‐cycle curves of Li_2_S−Li_2_O−LiI cell, and cycling (50 mA/g) of Li_2_S−C and Li_2_S−Li_6_PS_5_Cl−C electrodes. Reproduced with permission from [14]. Copyright 2016, American Chemical Society. (c) Schematic of an Li_2_S−Li_2_O−LiI‐based all‐solid‐state cell and its first‐cycle charge–discharge curves. Reproduced with permission from [95]. Copyright 2023, Wiley‐VCH.

As a cathode active material for Li‐S batteries, Li_2_S has a high activation overpotential during the first charge process, with the core bottleneck being slow decomposition kinetics. Recent studies have found that the introduction of catalytic media can significantly alleviate this kinetic bottleneck. Using diphenyl ditelluride (DPDTe) as a redox medium can reduce the initial charging voltage of the Li_2_S cathode to 2.4 V and achieve a specific capacity of 706.8 mAh·g^−1^ (calculated based on the mass of Li_2_S) after 360 cycles, proving that this strategy can effectively reduce the reaction activation energy barrier and improve the Li_2_S utilization rate (Figure 11a) [93]. In addition, constructing a WS_2_ phase with mixed ionic‐electronic conductivity can promote the reversibility of polysulfide reactions and inhibit the accumulation of harmful by‐products, thereby improving interfacial stability and battery cycle performance [93, 94].

In terms of nanocomposite electrode design, uniformly compounding Li_2_S, Li_6_PS_5_Cl (with a particle size of approximately 4 nm) and carbon materials can not only construct a complete ionic/electronic conduction network but also achieve a Li_2_S utilization rate of 71% and a reversible capacity of 830 mAh·g^−1^, with stable performance remaining after 60 cycles (Figure 11b) [14]. Another work developed a Li_2_S‐Li_2_O‐LiI cathode material, in which Li_2_O‐LiI forms nanoscale ion conduction channels during cycling, effectively activating Li_2_S and enabling the all‐solid‐state battery to achieve a high areal capacity of 10.6 mAh·cm^−2^ at 25°C (Figure 11c) [95]. The results show that regulating the Li_2_S decomposition pathway through catalytic design or interfacial engineering is a key strategy to improve its electrochemical activity and the overall performance of batteries.

### 7.4 Application‐Specific Requirements and Route Selection for Cathode‐Grade and Electrolyte‐Grade Li_2_S

Although Li_2_S is widely used in both Li−S batteries and sulfide solid electrolytes, these two application scenarios impose different requirements on its physicochemical properties. Therefore, green Li_2_S synthesis routes should not be evaluated only by yield, cost, and carbon footprint, but also by their ability to deliver application‐specific material quality.

For cathode‐grade Li_2_S used in Li−S batteries, the key requirement is to improve electrochemical accessibility and activation kinetics. Because Li_2_S suffers from poor intrinsic electronic/ionic conductivity and a high initial activation barrier, small particle size, high specific surface area, controllable morphology, and good compatibility with conductive carbon or catalytic hosts are highly desirable [4, 96]. Nanosized or porous Li_2_S particles can shorten Li^+^/electron transport pathways, enlarge the reaction interface, and facilitate the reversible conversion between Li_2_S and polysulfides/sulfur species. Therefore, cathode‐grade Li_2_S not only requires high chemical purity but also demands morphology control, dispersibility, and interfacial compatibility with conductive/catalytic matrices [97, 98, 99].

In contrast, electrolyte‐grade Li_2_S used for sulfide solid electrolyte synthesis places stricter emphasis on chemical purity, stoichiometric accuracy, crystallinity, and impurity control. Oxygen‐containing lithium impurities, including Li_2_O, Li_2_CO_3_, Li_2_SO_3_, Li_2_S_2_O_3_, and Li_2_SO_4_, as well as residual metal oxides, inorganic salts, solvent residues, and moisture, may disrupt sulfide electrolyte formation and degrade ionic conductivity or interfacial stability [36, 37]. Therefore, electrolyte‐grade Li_2_S should possess highly consistent composition, low oxygen and moisture content, low residual salt/metal impurity levels, and stable batch‐to‐batch reproducibility. Unlike cathode‐grade Li_2_S, an excessively high surface area is not always preferred for electrolyte precursors because it may increase moisture sensitivity and surface oxidation during storage or handling.

Based on these different requirements, the suitability of green synthesis routes should be considered in an application‐oriented manner (Table 8). Low‐temperature solid‐state routes are attractive for low‐cost and scalable production, but their application in electrolyte‐grade Li_2_S requires strict control over oxygen‐containing intermediates such as Li_2_SO_3_ and Li_2_S_2_O_3_. If particle size and composite dispersion can be controlled, such routes may be more suitable for cathode‐grade Li_2_S. Metallothermic reduction can provide highly crystalline Li_2_S through a solvent‐free pathway, but residual MgO/Al_2_O_3_ and unreacted reductants must be efficiently removed for electrolyte‐grade applications. Solution‐based metathesis offers advantages in purity control, crystallization regulation, and continuous production, making it particularly promising for electrolyte‐grade Li_2_S when residual salts and solvents are effectively eliminated. Meanwhile, its precipitation/crystallization nature also allows particle‐size engineering, which can be beneficial for cathode‐grade Li_2_S if nanoscale morphology and conductive‐host integration are further optimized.

**TABLE 8:** Application‐specific requirements and suitable green synthesis routes for cathode‐grade and electrolyte‐grade Li_2_S.

Application scenario	Key material requirements	Sensitive impurities/limitations	Preferred Li_2_S features	More suitable green synthesis routes	Main reason
Cathode‐grade Li_2_S for Li−S batteries	Small particle size, high specific surface area, controllable morphology, good dispersibility, compatibility with carbon/catalytic hosts	Insulating surface oxides, residual carbonates, large agglomerates, and poor conductive contact	Nanosized or porous Li_2_S with high electrochemical accessibility and low activation barrier	Low‐temperature solid‐state synthesis; solution‐based metathesis with controlled precipitation	These routes allow particle‐size/morphology regulation and can facilitate composite cathode construction.
Electrolyte‐grade Li_2_S for LPS/LPSCl/LGPS‐type sulfide electrolytes	High chemical purity, accurate stoichiometry, stable crystallinity, low oxygen/moisture content, low residual metal/salt impurities, batch reproducibility	Li_2_O, Li_2_CO_3_, Li_2_SO_3_, Li_2_SO_4_, MgO/Al_2_O_3_, NaCl/Na_2_SO_4_, solvent residues, moisture	High‐purity Li_2_S with controlled composition and minimal impurity carryover	Solution‐based metathesis; solvent‐free low‐temperature synthesis after sufficient purification; metallothermic reduction after efficient oxide removal	These routes can provide high‐purity Li_2_S if residual salts, solvents, oxygen‐containing intermediates, or oxide by‐products are strictly controlled.
Dual‐use Li_2_S platform	Balanced purity, morphology control, scalable production, low cost, low carbon footprint	Trade‐off between high surface area and air/moisture sensitivity; trade‐off between purity and processing cost	Tunable Li_2_S with route‐dependent particle size, purity, and surface chemistry	Hybrid routes combining low‐temperature synthesis, crystallization control, and closed‐loop purification	Application‐specific post‐treatment can tailor Li_2_S toward either cathode or electrolyte use.

Therefore, future green Li_2_S manufacturing should move from a route‐centered evaluation toward an application‐specific quality‐control framework. Cathode‐grade Li_2_S should prioritize electrochemical activation, interfacial accessibility, and composite‐process compatibility, whereas electrolyte‐grade Li_2_S should prioritize chemical purity, stoichiometric precision, impurity suppression, and batch reproducibility.

## 8 Current Challenges and Controversies

### 8.1 Balancing the Technical and Economic Feasibility of by‐Product Recycling and Utilization

Although green synthetic routes have significantly reduced carbon emissions and energy consumption in the Li_2_S preparation process, the efficient recycling and utilization of by‐products still face the challenge of balancing technical and economic feasibility. In the LiOH‑S low‐temperature solid‐state reaction system, the main by‐products include lithium sulfite (Li_2_SO_3_), lithium thiosulfate (Li_2_S_2_O_3_), and water, with an initial lithium conversion rate of only approximately 57%. A large amount of lithium resources exist in the form of by‐products, making it imperative to develop an efficient recovery mechanism to improve atom economy [43]. Therefore, by‐products should not be discussed only in terms of their potential value, but should be classified according to their chemical identity, formation source, environmental risk, separation difficulty, and closed‐loop utilization pathway. In addition to the representative by‐products such as MgO from magnesiothermal reduction and NaCl/Na_2_SO_4_ from metathesis reactions, other secondary streams, including unreacted precursors, oxygen‐containing lithium intermediates, sulfur‐containing gases, residual carbon, washing‐derived soluble salts, spent solvents, and gas‐phase products generated during low‐temperature or solvent‐free reactions, should also be considered [100].

For low‐temperature solid‐state reactions, Li‐containing intermediates such as Li_2_SO_3_ and Li_2_S_2_O_3_ should not be regarded simply as impurities, because they also represent unrecovered lithium resources. Their controlled thermal conversion, reductive transformation, or reintroduction into upstream precursor streams can improve lithium recovery efficiency and atom economy. Possible sulfur‐containing gases generated under non‐ideal reaction conditions should be captured through alkaline scrubbing or related absorption strategies and converted into manageable sulfide, sulfite, sulfate, or thiosulfate salts [101]. For thiourea‐assisted solvent‐free synthesis, NH_3_ and CO_2_ gas streams should also be incorporated into the closed‐loop design, where NH_3_ can be absorbed as ammonium salts, and CO_2_ can be captured or mineralized when feasible [33].

For magnesiothermal reduction, MgO is the dominant solid by‐product. Although MgO is chemically stable and relatively benign, its practical value depends on phase purity, particle‐size distribution, and separation cost. High‐purity MgO can be reused as a ceramic additive, separator coating component, or cathode‐stabilizing filler. A more complete circular strategy would further require MgO‐to‐Mg regeneration using low‐carbon energy input, thereby constructing a Mg/MgO circular loop [102, 103]. Therefore, MgO should be regarded not merely as an inert solid waste but as a potential node in metallothermal process circularity [102].

Although the metathesis reaction route can realize “solvent‐induced crystallization” via low‐boiling anti‐solvents and recycle solvents through vacuum distillation to construct a closed‐loop continuous production system, this process has high requirements for equipment tightness, solvent purity and energy consumption control, which may offset part of the cost advantages (Table 9) [26]. In addition to solvent recovery, closed‐loop salt management is also required because the major secondary streams of solution‐based metathesis include soluble salts such as NaCl, Na_2_SO_4_, LiNaSO_4_, residual lithium/sodium salts, salt‐rich mother liquor, and spent solvents. These streams should be managed through solvent distillation, mother‐liquor recycling, fractional crystallization, membrane separation, and closed‐loop washing. Recovered NaCl and Na_2_SO_4_ can be reused as industrial salts when purity requirements are satisfied, while lithium‐containing salts can be converted back into reusable lithium precursors. Therefore, the sustainability of metathesis routes depends not only on solvent recovery but also on closed‐loop salt crystallization, washing‐liquid reuse, and impurity accumulation control.

**TABLE 9:** Closed‐loop management strategies for representative by‐products and secondary waste streams in Li_2_S synthesis routes.

Synthesis route	Representative by‐products/secondary streams	Main concern	Separation or capture strategy	Closed‐loop management pathway	Key limitation
High‐temperature carbothermal reduction	CO/CO_2_, SOx, residual carbon, unreacted Li_2_SO_4_, Li_2_CO_3_, Li_2_O, Li_2_SO_3_/Li_2_SO_4_ surface impurities	High carbon emission, gas‐treatment burden, impurity accumulation, and lithium loss	CO_2_ capture, SOx scrubbing, solid impurity separation, washing, and lithium salt recovery	Captured CO_2_/SOx streams can be treated or mineralized; lithium‐containing residues can be redirected into upstream lithium salt feedstocks; residual carbon may be recovered or combusted with emission control.	High furnace energy and complex gas/solid waste treatment limit closed‐loop efficiency
Low‐temperature LiOH−S solid‐state reaction	H_2_O, unreacted LiOH/S, Li_2_SO_3_, Li_2_S_2_O_3_, possible H_2_S/SOx under side reactions	Incomplete lithium conversion, oxygen‐containing impurities, and possible sulfur‐containing gas emission	Controlled thermal conversion, sulfur‐ratio optimization, recovery of unreacted Li species, alkaline gas scrubbing	Li‐containing intermediates can be converted back toward Li_2_S; unreacted LiOH can be recycled into the feed stream; captured sulfur species can be converted into sulfite, sulfate, thiosulfate, or sulfide salts.	Requires precise control of intermediate conversion and gas‐capture units
Thiourea‐assisted solvent‐free route	NH_3_, CO_2_, H_2_O, possible HNCN/cyanamide‐derived intermediates, residual LiOH, Li_2_CO_3_, Li‐containing residues	Gas emission, odor/toxicity concern, lithium utilization loss, carbonate contamination	Acid absorption of NH_3_, CO_2_ capture or alkaline absorption, controlled gas exhaust, and recovery of residual lithium salts	NH_3_ can be converted into ammonium salts; residual lithium species can be recycled into precursor streams; CO_2_ can be captured or mineralized where feasible	Gas‐treatment cost and purity control of recycled lithium species
Boron‐assisted low‐temperature sulfidation	Boron oxides/borates, oxygen‐containing residues, unreacted oxide/sulfide precursors	Accumulation of boron‐containing residues and oxygen impurities	Solid separation, washing, phase‐purity control, borate recovery	Boron‐containing residues may be reused as oxygen scavengers or converted into borate products if purity permits	Recycling feasibility depends on boron residue composition and separation efficiency
Magnesiothermal reduction	MgO, residual Mg, Mg(OH) _2,_ or Mg salts generated during washing, and unreacted lithium salts	Solid by‐product accumulation, Mg‐resource loss, washing‐derived waste	Solid−solid separation, controlled washing/filtration, particle‐size classification, MgO purification	High‐purity MgO can be reused as a ceramic additive, separator coating component, cathode‐stabilizing filler, or regenerated to Mg using low‐carbon energy	MgO value depends on purity, particle size, and energy demand of MgO‐to‐Mg regeneration
Aluminothermal reduction	Al_2_O_3_, residual Al, aluminum hydroxides, or soluble Al salts after washing, and unreacted lithium salts	Solid residue accumulation, separation burden, and possible washing wastewater	Solid separation, washing, filtration, particle‐size control, Al_2_O_3_ purification	Al_2_O_3_ can be reused as a ceramic filler, separator coating component, or catalyst/support material if purity and morphology meet requirements.	Direct reuse requires strict control of Al_2_O_3_ purity, particle size, and surface chemistry.
Solution metathesis	NaCl, Na_2_SO_4_, LiNaSO_4_, NaNO_3_/NaX depending on lithium salt, residual Na_2_S, residual Li salts, spent solvent, salt‐rich mother liquor	Salt‐rich wastewater, solvent loss, impurity carryover, and incomplete Li recovery	Solvent distillation, mother‐liquor recycling, fractional crystallization, membrane separation, and closed‐loop washing	NaCl/Na_2_SO_4_ can be recovered as industrial salts; lithium‐containing salts can be converted back into reusable Li precursors; solvent can be recycled into the reaction system.	Requires high solvent recovery efficiency, controlled salt purity, and prevention of impurity accumulation
Solvothermal/solution‐assisted synthesis for sulfide electrolyte precursors	Residual solvents, LiCl/NaCl‐type salts, unreacted Li_2_S/P_2_S_5_/LiX, sulfur/phosphorus‐contain ing residues	Solvent management, salt contamination, sensitivity to moisture/oxygen	Solvent recovery, filtration, washing, low‐temperature drying, salt crystallization	Solvents can be recycled; residual lithium salts can be redirected into precursor preparation; recovered salts can be treated as industrial salts if sufficiently pure	Moisture sensitivity and solvent purity requirements increase process complexity

*Note*: The listed streams and management pathways are summarized based on the reaction characteristics and reported by‐product types of representative Li_2_S synthesis routes. They are intended as a conceptual framework for process design rather than experimentally validated closed‐loop schemes for every route. The specific by‐products depend on precursor selection, reaction atmosphere, conversion degree, and purification protocol.

In addition, some studies have attempted to convert the by‐product LiNaSO_4_ and unreacted Li_2_SO_4_ into Li_2_CO_3_ and Na_2_SO_4_ to realize resource reuse; however, the conversion steps increase the process complexity, and the economic feasibility is highly dependent on raw material price fluctuations and downstream market acceptance [27]. Therefore, how to control the additional treatment costs while ensuring a high recovery rate is a core problem that must be solved for the current green synthesis processes to move toward industrialization.

### 8.2 Challenges in the Treatment of Oxidation Products from Non‐Carbon Reducing Agents

Although non‐carbon reduction strategies avoid CO_2_ emissions from the source, there are still controversies regarding the environmental compatibility and high‐value treatment pathways of their oxidation by‐products. The MgO produced by the magnesiothermal reduction method has stable chemical properties, but its large‐scale high‐value utilization pathway remains to be developed. If it is not effectively resource‐treated, it may bring pressure on solid waste management [23]. Similarly, the Al_2_O_3_ generated by aluminothermal reduction can theoretically be applied to battery separator coatings, but this application has strict requirements on product purity, particle size distribution, and surface characteristics. Al_2_O_3_ obtained from conventional reactions is difficult to meet the standards of electrode materials, limiting its direct recycling potential [79].

Although the metathesis reaction is touted as a “waste‐free” process, waste liquid or filter residue that requires treatment may still be generated in actual operation due to solvent residue, ionic impurities, or the accumulation of trace secondary salts. Notably, although these oxidation products do not have the properties of greenhouse gases, their long‐term environmental impact, disposal compliance, and potential secondary pollution risks have not been systematically evaluated. Especially in large‐scale industrial production scenarios, they may trigger new environmental regulatory pressures.

### 8.3 Multiphase Reaction Kinetics and Scale‐Up Effects in Industrial Production

**FIGURE 12:**
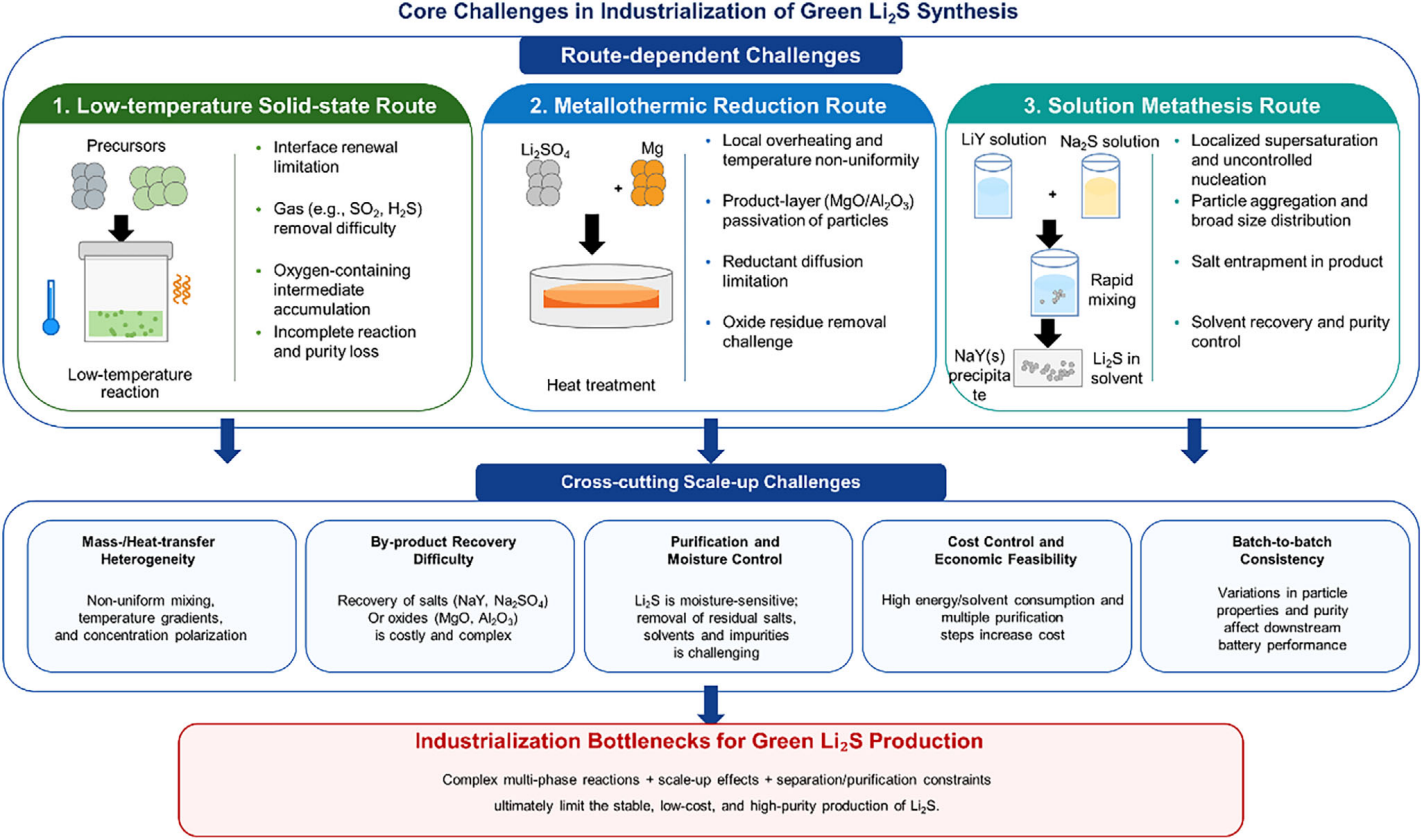
Schematic illustration of the key technical challenges and industrialization bottlenecks faced by green Li_2_S synthesis.

When scaling up from milligram‐scale laboratory verification to kilogram‐scale and even ton‐scale industrial production, green Li_2_S synthesis processes encounter significant scale‐up challenges (Figure 12). Although some green synthesis methods have demonstrated excellent performance in small‐scale tests, the scale‐up of Li_2_S synthesis systems remains constrained by multi‐scale mass transfer limitations and interfacial contact efficiency [104, 105]. Solid‐solid reactions are extremely sensitive to particle mixing uniformity, contact area, and heat transfer efficiency. To further clarify the scale‐up bottlenecks from a mechanistic perspective, the emerging Li_2_S synthesis routes should be analyzed as multiphase reaction systems rather than simple process flows. In low‐temperature solid‐state reactions, the overall conversion is governed by the formation, renewal, and replenishment of solid−solid reaction interfaces between lithium and sulfur‐containing precursors. Although mechanical activation can generate fresh contact interfaces and local reaction hotspots, the interfacial replenishment rate decreases markedly during scale‐up because particle mixing, heat transfer, and gas removal become spatially non‐uniform. This may lead to unreacted cores, local accumulation of oxygen‐containing intermediates, and heterogeneous lithium conversion across the reactor [106].

Metallothermic reduction involves even stronger multiphase coupling. For magnesiothermal or aluminothermal reduction, the reaction begins at the contact interface between the metal reductant and lithium/sulfur‐containing precursor, accompanied by rapid heat release and formation of oxide by‐products such as MgO or Al_2_O_3_. These oxide products may form a shell‐like barrier around reactive particles, limiting further contact between fresh reductant and precursor. Therefore, the apparent reaction rate is not controlled only by thermodynamics or electron transfer, but also by heat dissipation, product‐layer diffusion, local melting or vapor‐phase transport of the reductant, and the continuous renewal of reactive interfaces. During scale‐up, insufficient heat management or poor precursor mixing may amplify local overheating, incomplete reduction, and batch‐to‐batch heterogeneity [107].

In contrast, solution‐based metathesis is dominated by mixing, precipitation, and crystallization kinetics. The formation of Li_2_S is driven by its extremely low solubility, but rapid ion exchange can generate localized supersaturation before macroscopic mixing is completed. Once local supersaturation exceeds the nucleation threshold, burst nucleation, particle aggregation, and salt entrapment may occur, resulting in a broad particle‐size distribution and reduced product purity. Therefore, reactor design for metathesis routes should focus on micromixing intensity, feeding sequence, residence‐time distribution, antisolvent addition rate, crystallization temperature, and mother‐liquor circulation. These parameters determine whether precipitation proceeds through uniform nucleation and controlled growth or through uncontrolled local crystallization [108]. In situ XRD studies show that the reaction rate of particles without direct contact is significantly reduced, and the initial reaction kinetics are dominated by the material stacking mode, a characteristic that is difficult to accurately reproduce in large reactors.

Although low‐temperature air‐stable synthesis can be achieved at temperatures below 200°C, parameters such as carrier gas flow rate, moisture control, and sulfur excess ratio are prone to gradient distribution during scale‐up, affecting product consistency and lithium conversion rate. More importantly, the cost advantages of existing green processes (with self‐made Li_2_S costing approximately ∼$52.0 per kilogram) are largely derived from idealized small‐scale test data. Upon actual scale‐up, costs such as energy consumption, equipment depreciation, labor, and by‐product treatment are likely to increase significantly, diminishing their economic competitiveness [43]. Therefore, establishing a scalable engineering model, optimizing reactor design, and carrying out systematic pilot verification are the keys to breaking through the last mile of green Li_2_S synthesis from “laboratory feasible” to “industrially applicable”.

## 9 Future Research Directions

### 9.1 Exploration of New Reduction Pathways Driven by Photo/Electrocatalysis

**FIGURE 13:**
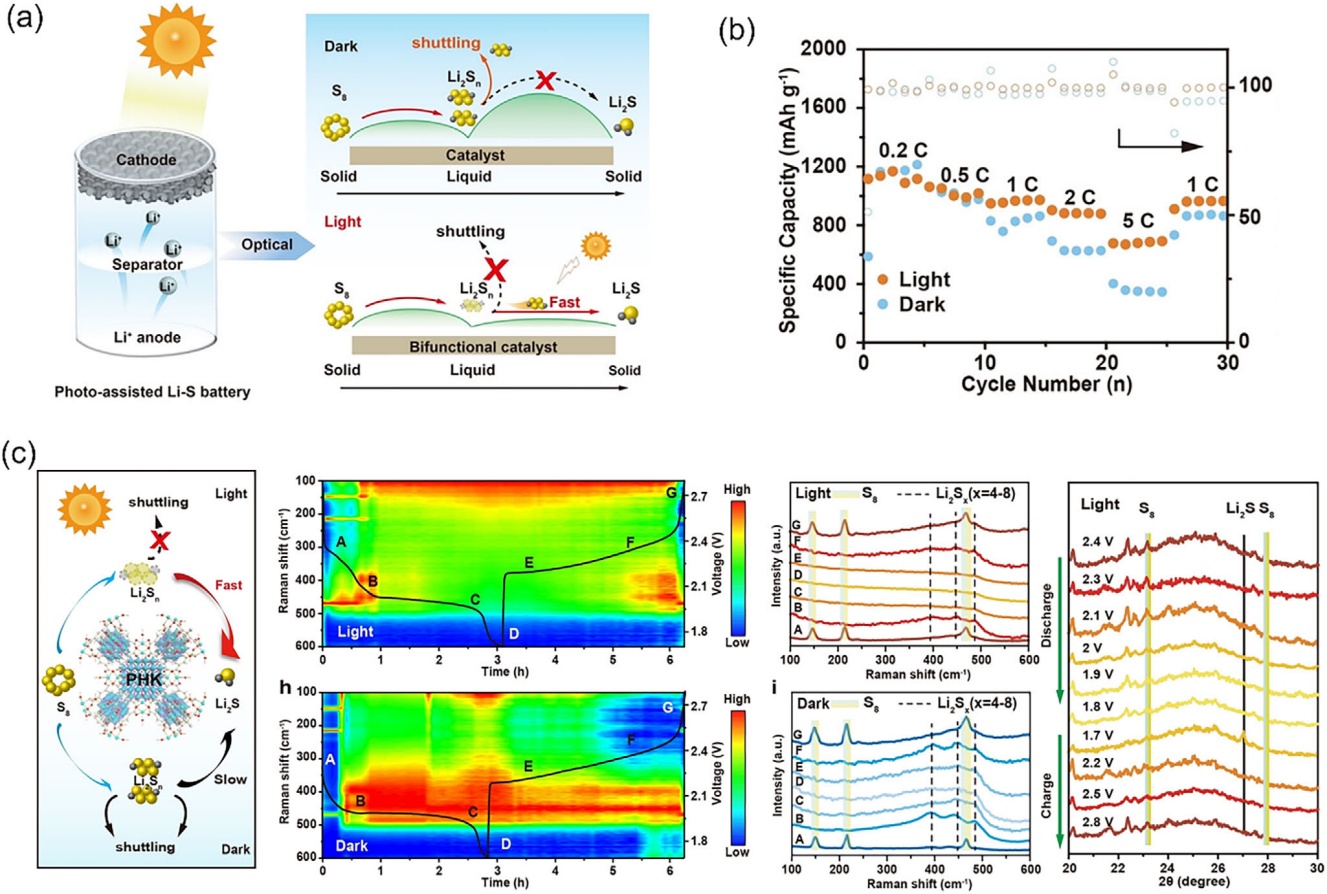
(a,b) Discharge processes of Li–S batteries without and with illumination, schematic of PA‐LSB, selective catalysis mechanism; (c) in situ Raman contour plots and spectra with/without illumination, and ex situ XRD patterns with illumination. Reproduced with permission from [112]. Copyright 2024, Wiley‐VCH.

In recent years, photocatalytic and electrocatalytic technologies have demonstrated great potential in green synthesis, offering new approaches to the low‐carbon preparation of Li_2_S [99, 109, 110, 111]. Existing research has confirmed that visible light‐driven catalysis systems can significantly reduce the reaction activation energy and enhance reaction selectivity. In Li‐S battery systems, perovskite quantum dot‐loaded metal‐organic framework materials as cathodes can accelerate the redox reaction of sulfur species under light irradiation, realize the selective catalysis of the liquid‐solid conversion stage of polysulfides, enable the battery to obtain a reversible capacity of 679 mAh·g^−1^ at a 5 C rate, with a capacity decay rate of only 0.022% per cycle after 1500 cycles, and simultaneously have photo‐charging capability (Figure 13a–c). This provides a direct experimental basis for the introduction of photocatalytic pathways in Li_2_S synthesis [112].

TiO_2_/CoFe_2_O_4_ nanocomposites exhibit an excellent photocatalytic reduction performance and stability with a reduction rate of over 94% for 4‐nitrophenol within 35 min under visible light, and their electron transfer mechanism can provide an important reference for sulfur source reduction [113]. GaN‐based materials have shown outstanding performance in fields such as photocatalytic water splitting, CO_2_ reduction, and nitrogen fixation due to their wide light absorption range, high‐efficiency charge separation capability, and good chemical stability. Their catalytic activity can be further improved through nanostructure design, doping, or heterojunction construction, which is expected to be extended to the field of sulfide photoreduction synthesis [114]. At the same time, metal sulfides themselves have tunable electronic structures and abundant active sites, which can be used for reduction reactions in artificial photosynthesis. While their application in solar‐driven Li_2_S synthesis remains restricted by the spectral response scope and active site density, breakthroughs are expected to be achieved via interfacial engineering and defect modulation [115].

### 9.2 Artificial Intelligence‐Assisted Optimization of Reaction Conditions

**FIGURE 14:**
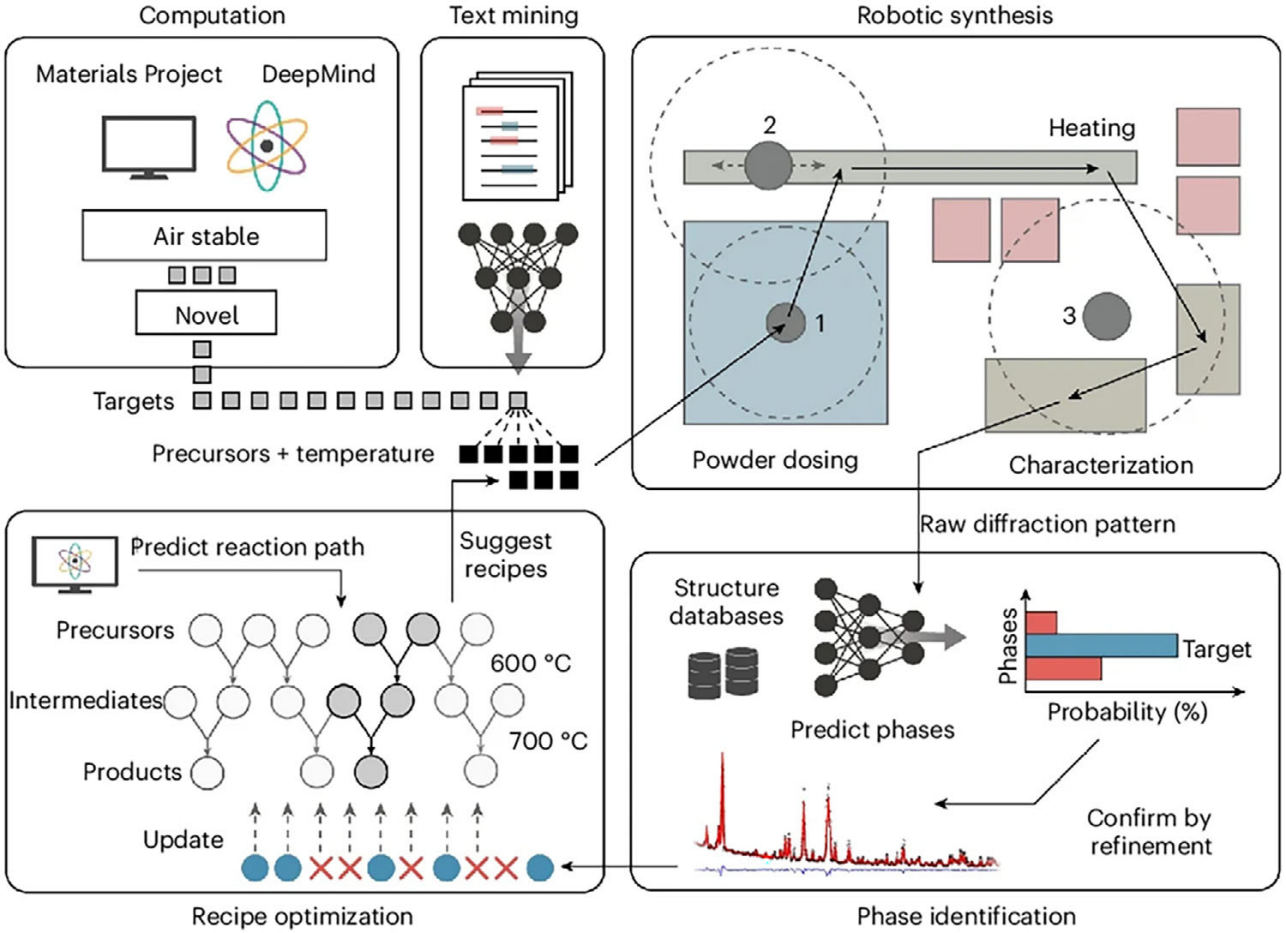
Schematic of autonomous materials discovery with A‐Lab, integrating computation, text mining, robotic synthesis, recipe optimization, and phase identification to optimize reaction pathways. Reproduced with permission from [116]. Copyright 2025 Springer Nature.

With the rapid development of computational chemistry and machine learning technologies, the role of artificial intelligence (AI) in reaction pathway prediction and process parameter optimization has become increasingly prominent (Figure 14) [116]. Current green Li_2_S synthesis involves a multi‐variable coupling system including temperature, material ratio, solvent system, and catalyst type, and the traditional trial‐and‐error method is inefficient. DFT‐based first‐principles calculations have been successfully used to identify key descriptors of the sulfur reduction reaction (SRR), such as the adsorption energy of LiS intermediates, and based on this, Cu@CN single‐atom catalysts have been screened out with the lowest overpotential (0.426 V) [117]. Similarly, PtCo alloys exhibit the optimal sulfur species adsorption energy and reaction kinetics due to their balanced d‐band center, confirming that electronic structure parameters can be used as effective prediction indicators for catalytic activity [118].

The aforementioned theoretical results have laid a solid foundation for the construction of AI training datasets. In the future, by integrating in situ characterization data (in situ XRD, Raman) and high‐throughput experimental results, a multi‐objective optimization model of “reaction conditions‐product purity‐energy consumption” can be established to realize the intelligent regulation of low‐temperature solid‐state reactions or metathesis pathways [119]. Especially in non‐carbon reduction systems such as magnesiothermal reduction or Na_2_S metathesis reactions, AI can quickly identify the optimal reaction window, avoid the generation of by‐products, improve atom economy and greatly enhance the efficiency of process development [26, 120].

### 9.3 Integration of Green Processes for Closed‐Loop Production Systems

**FIGURE 15:**
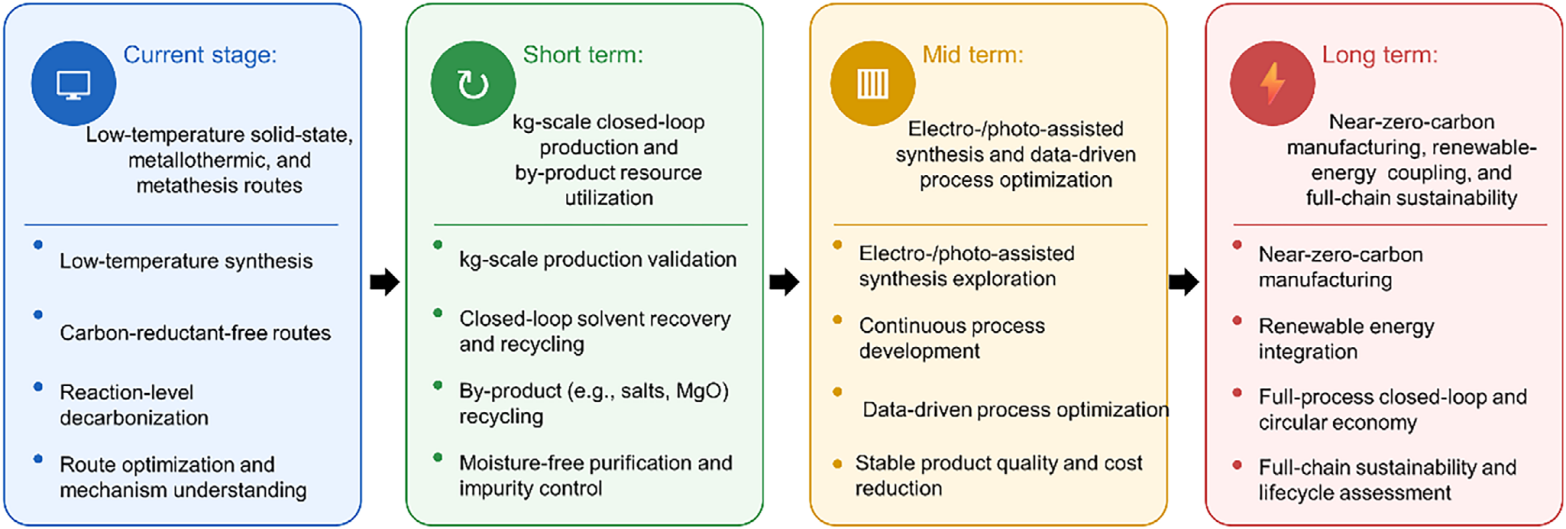
Future development roadmap for green and low‐carbon Li_2_S synthesis technologies.

Constructing a closed‐loop, continuous, and low‐waste integrated production process is the core path for the industrialization of green Li_2_S synthesis (Figure 15). Existing studies have proposed a new waste‐free method for synthesizing Li_2_S: directly precipitating crystalline Li_2_S at room temperature through the metathesis reaction between lithium salts and sodium sulfide, and realizing efficient solvent separation and recycling using low‐boiling anti‐solvents. The entire process can be operated in a closed‐loop continuous system, avoiding the emission of harmful waste, and the resulting product exhibits excellent performance in batteries, providing a classic example for closed‐loop processes.

In the future, it is necessary to further integrate the by‐product recovery module. For example, aiming at MgO generated in magnesiothermal reduction or sodium salt by‐products in metathesis reactions, develop in situ regeneration or resource high‐value utilization technologies to balance technical and economic feasibility. At the same time, drawing on the mechanism of enhanced electron‐hole separation by the strong electric field at the air‐water interface of microdroplet reactors, a microfluidic continuous synthesis device can be designed to improve mass transfer efficiency and reduce reaction energy consumption. In addition, coupling photo/electrocatalytic units with solvent recovery systems to form an integrated “reaction‐separation‐regeneration” platform can not only reduce raw material consumption but also significantly lower the carbon footprint. If such integrated systems are coupled with renewable energy sources such as photovoltaic power for power supply, true zero‐carbon manufacturing of Li_2_S synthesis will be realized, fully aligning with the global carbon neutrality strategic goals.

## Author Contributions

**Aiping Peng**: Writing – original draft, Investigation, Formal analysis, Data curation, Conceptualization. **Xingtao Qi**: Writing – original draft, Data curation, Formal analysis. **Lingfeng Zhu**: Conceptualization, Data curation, Formal analysis, Supervision, Resources, Project administration, Methodology, Writing – review & editing. **Ningrui Zhan**: Data curation. **Yaohui Qu**: Visualization, Funding acquisition. **Hui Li**: Data curation. **Hai Zhang**: Investigation. **Fan Wang**: Resources. **Ze Zhang**: Visualization, Funding acquisition. **Xuewen Wang**: Supervision, Writing – review & editing. **Zhenyu Yang**: Supervision, Project administration, Funding acquisition, Writing – review & editing. **Tianyi Ma**: Supervision, Writing – review & editing.

## Conflicts of Interest

The authors declare no conflict of interest.

## Data Availability

Data sharing not applicable to this article as no datasets were generated or analysed during the current study.
